# Hepatocyte‐Specific HuR Protects Against Acetaminophen‐Induced Liver Injury in Mice

**DOI:** 10.1111/jcmm.70246

**Published:** 2024-11-29

**Authors:** Linlin Lu, Jicui Chen, Hui Jiang, Ni Li, Xiaojie Li, Yinghao Liu, Chen Zong

**Affiliations:** ^1^ Institute of Medical Sciences, the Second Hospital, Cheeloo College of Medicine Shandong University Jinan Shandong China; ^2^ Department of Blood Transfusion Qilu Hospital of Shandong University Jinan China; ^3^ Department of Cell Biology Shandong University School of Medicine Jinan China

**Keywords:** APAP‐induced liver injury, autophagy, hepatocyte proliferation, HuR, oxidative stress

## Abstract

Acetaminophen (APAP) overdose is a major cause of drug‐induced liver injury (DILI) in many countries. Hepatocyte proliferation, autophagy and antioxidant capacity are crucial to the prognosis of APAP‐induced liver injury, but the underlying mechanisms are not fully understood. Here, we found that human antigen R (HuR) protein expression was markedly increased in the model of APAP‐induced liver injury, and conditional hepatocyte‐specific HuR knockout aggravated APAP‐induced liver injury in mice. Further investigation of the underlying mechanisms of HuR's protective effects showed that conditional hepatocyte‐specific HuR knockout reduced the protein expression of cyclin A1, cyclin B1, cyclin D1, CDK2, ATG3, ATG5, ATG7 and NRF2 in mice, reducing hepatocyte proliferation, autophagy and antioxidant capacity. Mechanistically, HuR could physically associate with the 3′‐untranslated regions (UTRs) of cyclin A1, cyclin B1, cyclin D1, Cdk2, Atg3, Atg5, Atg7 and Nrf2 mRNAs, thereby regulating their translation. These findings suggest that HuR attenuates APAP‐induced liver injury by regulating hepatocyte proliferation, autophagy and antioxidant capacity.

## 1 Introduction

Drug‐induced liver injury (DILI) is one of the most common and serious adverse drug reactions, which can lead to acute liver failure and even death, according to the World Health Organisation [1]. As a commonly used clinical drug, acetaminophen (APAP) is safe and effective at normal doses, but excessive doses may lead to DILI and acute liver failure. In fact, APAP‐induced hepatotoxicity remains a major cause of DILI and acute liver failure in many countries [2, 3]. It has been admitted that the APAP‐induced hepatotoxicity consists of multi‐stages and multi‐signalling pathways, including APAP metabolism, oxidative stress, hepatocyte necrosis, autophagy, and compensatory liver repair and regeneration.

Excessive APAP is metabolised by cytochrome P4502E1 (CYP2E1) to form excessive N‐acetyl‐p‐benzoquinone imine (NAPQI), which is the main source of hepatotoxicity [4]. The highly reactive NAPQI further binds to hepatic glutathione (GSH) and intracellular proteins, especially mitochondrial proteins [5]. This leads to mitochondrial dysfunction and oxidative stress, ultimately hepatocyte necrosis [4]. In response to liver injury, the liver could regenerate and repair liver injury through compensatory hepatocyte proliferation [6]. Therefore, hepatocyte proliferation plays an important role in the prognosis of APAP‐induced liver injury. It has been reported that Parkin knockout mice were protected against APAP‐induced liver injury by increasing hepatocyte proliferation [7]. APAP overdose leads to mitochondrial damage and accumulation of APAP‐protein adducts (APAP‐ADs) in hepatocytes, resulting in hepatocyte necrosis and injury [8, 9]. Therefore, it is not surprising that autophagy, as a protective mechanism for maintaining cellular homeostasis, is activated in response to severe cellular stress induced by APAP. It was reported that Torin 1 mitigated APAP‐induced hepatotoxicity by inducing autophagy to remove damaged mitochondria and deleterious APAP‐Ads [10]. The major regulator of the antioxidant defence system in the body is the nuclear factor erythroid 2‐related factor 2 (NRF2) [11, 12, 13], which is likely activated by oxidative/electrophilic stress. Activated NRF2 dissociates from Kelch‐like ECH‐related protein 1 (KEAP1), translocated to the nucleus and activated antioxidant response element (ARE)‐responsive gene expression. The antioxidant enzymes activated by NRF2 include haeme oxygense‐1 (HO‐1), NADPH: quinone oxidoreductase 1 (NQO‐1), catalase (CAT), glutathione peroxidase (GPX), superoxide dismutase (SOD) and glutamate cysteine ligase (GCL) [14, 15], which catalyses the rate‐limiting step in GSH synthesis. Limonin has been reported to ameliorate APAP‐induced hepatotoxicity by activating the NRF2 antioxidant pathway [16].

Human antigen R (HuR) protein is a member of the embryonic lethal aberrant vision (ELAV) family, which is widely expressed in mammalian cells. HuR protein plays an important biological role, involved in the regulation of many cellular functions (proliferation, apoptosis, senescence, etc.) and the occurrence and development of many diseases, such as cancer [17]. It was reported that HuR regulated cell cycle–dependent stabilisation of mRNAs encoding cyclins A and B1 in human colorectal carcinoma RKO cells [18]. In addition, HuR was reported to regulate the protein expression of ATG7 and ATG16L1 and mediate autophagy in HK‐2 cells [19]. HuR was also reported to enhance Nrf2‐mRNA maturation and promote its nuclear export in HEK293T cells [20]. However, the effects of HuR in APAP‐induced liver injury and underlying mechanisms remain to be studied.

In the current study, we utilised a conditional hepatocyte‐specific HuR knockout mouse (cKO) to evaluate the effects of HuR in APAP‐induced liver injury. We found that HuR combined directly with the 3′UTRs of cyclin A1, cyclin B1, cyclin D1, Cdk2, Atg3, Atg5, Atg7 and Nrf2 mRNAs in mice, thereby regulating their translation. Our results thus revealed that conditional hepatocyte‐specific HuR knockout aggravated APAP‐induced liver injury by regulating hepatocyte proliferation, autophagy and antioxidant capacity in mice.

## 2 Materials and Methods

### 2.1 Ethics Statement

Animal experiments were carried out in accordance with the Principles of Laboratory Animal Care established by the National Institutes of Health. All experiments were approved by the Institutional Animal Care and Use Committee of Shandong University (approval No. KYKK‐2021 (KJ) A‐0090).

### 2.2 Animals

HuR floxed mice [21] and Albumin (Alb)‐Cre mice were gifted by professor Wang Wengong. C57BL/6J mice were purchased from Jinan Pengyue Experimental Animal Breeding Co. Ltd. Conditional hepatocyte‐specific HuR knockout mice (cKO) were generated by crossbreeding HuR^flox/flox^ mice with Alb‐Cre mice. HuR^flox/flox^/Cre^−^ mice were used as controls (CTR). All mice were housed in room temperature (~24°C) on a 12 h light/12 h dark cycle and were fed ad libitum and maintained on a specific pathogen free (SPF) status, except in the fasting period, during which just water was freely accessed. To induce liver injury, 8‐ to 10‐week‐old conditional hepatocyte‐specific HuR knockout male mice and their control littermates were fasted overnight before intraperitoneal (IP) administration of APAP (300 mg/kg; A800441, Macklin, Shanghai, China). For autophagy inhibition, mice were treated (IP) with chloroquine (CQ, 60 mg/kg; C843545, Macklin, Shanghai, China) simultaneously with APAP for 12 h. Then, mice were sacrificed under isoflurane inhalation (1.4%) followed by cervical dislocation at 0, 6 or 12 h post‐APAP. The male CTR and cKO mice with similar body weight and healthy condition were selected for random grouping.

### 2.3 Cell Culture, Transfection of Small Interfering RNAs and Plasmids

Mouse hepatoma Hepa1‐6 cells were purchased from ATCC and grown in Dulbecco's modified Eagle's medium (DMEM), supplemented with 10% FBS and antibiotics at 37°C in a humidified atmosphere containing 5% CO_2_. Control siRNA (UUGUUCGAACGUGUCACGUUU) and HuR‐directed siRNA (AAGAGGCAAUUACCAGUUUCA) or pGL3 reporter plasmids were transfected using lipofectamine 3000 (Invitrogen) according to the manufacturer's instructions.

### 2.4 Western Blot Analysis and Antibodies

Western blot analysis was conducted according to the standard procedures. Polyclonal anti‐HuR (11910‐1), polyclonal anti‐CYP2E1 (19937‐1), polyclonal anti‐NRF2 (16396‐1), polyclonal anti‐PCNA (10205‐2), polyclonal anti‐CYCLIN B1 (55004‐1), polyclonal anti‐ CYCLIN D1 (26939‐1), polyclonal anti‐CDK2 (10122‐1), polyclonal anti‐CDK4 (11026‐1), polyclonal anti‐ATG3 (11262‐2), polyclonal anti‐ATG5 (10181‐2), polyclonal anti‐ATG7 (10088‐2), polyclonal anti‐ATG12 (11264‐1), polyclonal anti‐P62 (18420‐1), polyclonal anti‐LC3 (14600‐1) and monoclonal anti‐GAPDH (60004‐1) were from Proteintech. Polyclonal anti‐TUBB (YT4780) was from ImmunoWay. Polyclonal anti‐GCLM (BS7121), polyclonal anti‐GCLC (BS6008), polyclonal anti‐NQO‐1 (BS6833), polyclonal anti‐HO‐1 (BS3978), polyclonal anti‐SOD1 (BS79538), monoclonal anti‐SOD2 (BS79538), polyclonal anti‐CAT (BS79696) and polyclonal anti‐LAMIN B (AP6001) were from Bioworld. Polyclonal anti‐CYCLIN A1 (A14529) and secondary goat anti‐mouse (AS003) or rabbit (AS014) antibodies conjugated to horseradish peroxidase (HRP) were from Abclone.

### 2.5 Quantitative Real‐Time PCR (qPCR)

Total RNA was isolated from livers or cells using M5 total RNA extraction reagent (MF034‐01; TRIgent). Reverse transcription (RT) PCR was conducted using a Fastking RT Kit (KR116‐02; Tiangen) according to the manufacturer's instructions. Quantitative real‐time PCR (qPCR) was conducted using a SuperReal PreMix Plus (SYBR green) Kit (Tiangen) according to the manufacturer's instructions. The relative gene expression was calculated by the ΔΔCq method. The primers used are listed in Table S1.

### 2.6 Preparation of Transcripts

cDNA was used as a template for PCR amplification to obtain DNA fragments, which were used as a template for PCR amplification to obtain RNA fragments. All 5′ primers were added with the T7 promoter sequence (CCAAGCTTCTAATACGACTCACTATAGGGAGA). The RNA fragments of cyclin A1‐3U (−32–188), cyclin B1‐3U (1–884), cyclin D1‐3U1 (−9–1003), cyclin D1‐3U2 (984–1905), cyclin D1‐3U3 (1885–2596), Cdk2‐3U1 (1–442), Cdk2‐3U2 (423–1114), Nrf2‐3U (−1–430), Atg3‐3U (−70–772), Atg5‐3U1 (−2–723), Atg5‐3U2 (703–1180), Atg7‐3U1 (−2–750) and Atg7‐3U2 (731–1559) were obtained. The primers used are listed in Table S2.

### 2.7 RNA Pull‐Down

Using the obtained DNA fragments as templates, biotin‐UTP was added to the reaction system and biotinylated RNA was transcribed using T7 RNA polymerase. 0.5 mg of purified biotinylated RNA was incubated with 200 μg of cell lysates for 30 min at room temperature; then, 20 μL of washed paramagnetic streptavidin‐conjugated Dynabeads (Dynal, Oslo) was added to the above reaction system and incubated for 30 min at room temperature, and the samples were washed twice with pre‐cooled PBS and analysed by Western blotting. The experiments were repeated thrice.

### 2.8 RNA Immunoprecipitation (IP) Assay

The RNA Immunoprecipitation (IP) Kit (P0101; Geneseed) was used for RNA IP assay. In brief, whole‐cell lysates were incubated with magnetic protein A + G beads pretreated with 5 μg of rabbit IgG (30,000‐0; Proteintech) or HuR (11,910‐1; Proteintech) antibody at 4°C overnight. RNA was isolated from the washed magnetic bead complexes, and cDNA was synthesised. RT‐qPCR was used to quantify the levels of different mRNA in magnetic bead complexes. The primers used are listed in Table S1.

### 2.9 Constructs

To construct pGL3‐derived reporter plasmids bearing the fragments of cyclin A1‐3U (−32–188), cyclin B1‐3U (1–884), cyclin D1‐3U1 (−9–1003), cyclin D1‐3U2 (984–1905), cyclin D1‐3U3 (1885–2596), Cdk2‐3U1 (1–442), Cdk2‐3U2 (423–1114), Nrf2‐3U (−1–430), Atg3‐3U1 (−70–289), Atg3‐3U2 (296–772), Atg5‐3U (−2–1003), Atg7‐3U1 (−2–750) and Atg7‐3U2 (731–1559), the primers used are listed in Table S3. These fragments were then digested with XbaI endonuclease and inserted into the pGL3‐promoter vector.

### 2.10 Double‐Luciferase Reporter Assays

Hepa1‐6 cells were seeded into 24‐well plates before transfection. Twenty‐four hours later, cells were transfected with control or HuR‐directed siRNAs for 48 h and then continued to be transfected with pGL3‐derived reporter plasmids depicted in Figure S10 plus a control plasmid vector (pRL‐TK). Twenty‐four hours later, luciferase activity was tested by using a double‐luciferase reporter assay kit (Trans Gen Biotech) according to the manufacturer's instructions. The experiments were repeated thrice.

### 2.11 Electron Microscopy

Fresh liver tissues were fixed in 2.5% glutaraldehyde in 0.01 M phosphate buffer (pH 7.0–7.4), and then electron microscopy analysis was performed by Wuhan Servicebio Technology Co. Ltd. (Wuhan, China).

### 2.12 Haematoxylin and Eosin (H&E) and Immunohistochemistry (IHC) Staining

Fresh liver tissues were fixed in 4% paraformaldehyde, and then haematoxylin and eosin (H&E) and immunohistochemical analysis were performed by Wuhan Servicebio Technology Co. Ltd. (Wuhan, China). Polyclonal anti‐HuR (11910‐1), polyclonal anti‐CYP2E1 (19937‐1) and polyclonal anti‐ki67 (28074‐1) antibodies were from Proteintech.

### 2.13 ALT and AST Measurements

Serum alanine aminotransferase (ALT) and aspartate aminotransferase (AST) were measured by Wuhan Servicebio Technology Co. Ltd. (Wuhan, China).

### 2.14 GSH Measurements

Hepatic GSH was measured using colorimetric analysis kits (Elabscience, Wuhan) according to the manufacturer's instructions.

### 2.15 ROS Measurements

Reactive oxygen species (ROS) were measured by using 2′,7′‐dichlorodihydrofluorescein diacetate (DCFH‐DA) (Elabscience, Wuhan, China) following the manufacturer's instructions. Briefly, after entering the cells, DCFH‐DA is hydrolysed into 2′,7′‐dichlorodihydrofluorescein (DCFH), which is oxidised to a fluorescent compound 2,7‐dichlorofluorescein (DCF) in the presence of ROS.

### 2.16 8‐OHdG Measurements

Hepatic 8‐OHdG was measured using mouse 8‐hydroxydeoxyguanosine (8‐OHdG) Elisa kit (Mlbio, Shanghai) according to the manufacturer's instructions.

### 2.17 Separation and Extraction of Nuclear Fractions

The nuclear proteins were collected by a nuclear and cytoplasmic protein extraction kit (Beyotime Biotechnology, Shanghai) according to the manufacturer's instructions.

### 2.18 Statistical Analysis

Data are expressed as the mean ± SD. Two‐tailed Student's *t*‐test or two‐tailed Mann–Whitney U test was used to analyse the significance of the data obtained from cells or animals, respectively. A value of *p* < 0.05 (*) was considered statistically significant.

## 3 Results

### 3.1 HuR Regulates Hepatocyte Proliferation, Autophagy and Antioxidant Capacity

To explore the function of HuR in APAP‐induced liver injury, we generated the conditional hepatocyte‐specific HuR knockout mice (cKO) by crossbreeding HuR^flox/flox^ mice with Alb‐Cre transgenic mice (Figure S1A) and genotypes were determined by PCR (Figure S1B). The specific knockout of HuR in hepatocytes was confirmed via qPCR (Figure S1C) and Western blot analyses (Figure S1D). As expected, the mRNA and protein levels of HuR were notably reduced in the liver but not in the other tissues of cKO mice compared with CTR (Figure S1C,D). Immunohistochemistry (IHC) staining further confirmed HuR deletion in the hepatocytes of cKO mice (Figure S1E).

**FIGURE 1:**
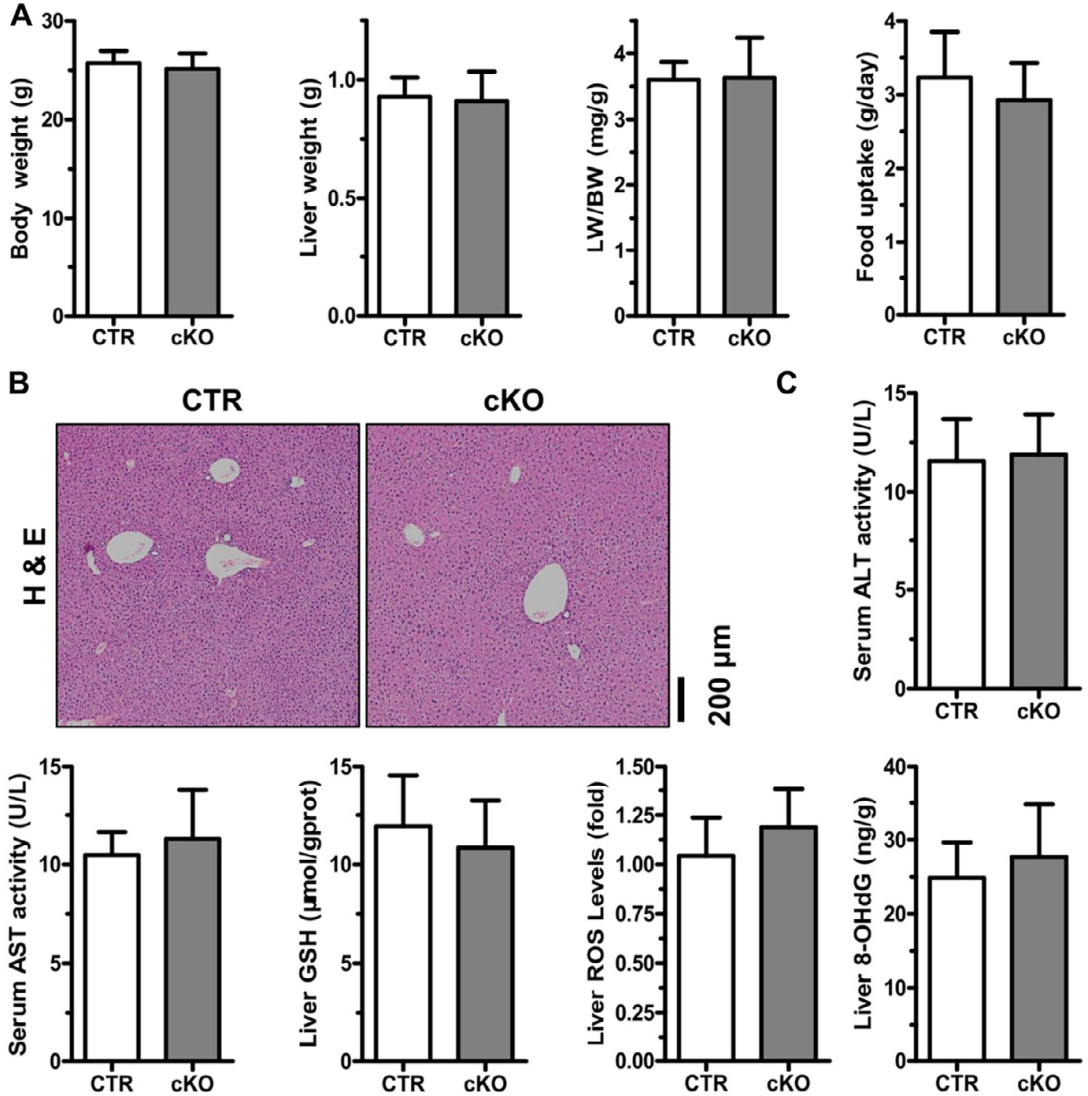
Conditional hepatocyte‐specific HuR knockout does not affect liver function. (A) Body weight, liver weight, the ratio of liver/body weight and food uptake of control (CTR, *n* = 5) and conditional hepatocyte‐specific HuR knockout mice (cKO, *n* = 5). (B) Representative photographs of haematoxylin and eosin (H&E) staining of liver sections are presented (CTR and cKO, *n* = 5) (scale bars, 200 μm). (C) Serum alanine aminotransferase (ALT), aspartate aminotransferase (AST) levels, hepatic glutathione (GSH), reactive oxygen species (ROS) and 8‐hydroxy‐2‐deoxyguanosine (8‐OHdG) levels were analysed (CTR and cKO, *n* = 5). Data are expressed as mean ± SD (blank columns, CTR; black columns, cKO).

The 8‐week‐old cKO and CTR mice were fed regular chow. cKO mice showed no significant changes in body weight, liver weight, the liver weight/body weight ratio and food uptake compared to CTR mice (Figure 1A). In addition, we found that the hepatocytes of cKO mice were similar to the CTR mice in morphology by H&E staining (Figure 1B). By serum and liver biochemistry analysis, the deletion of HuR did not affect the activity of ALT and AST in serum, the biomarkers of hepatocyte damage [22] and the levels of hepatic glutathione (GSH) and ROS (Figure 1C). Furthermore, we measured 8‐hydroxy‐2‐deoxyguanosine (8‐OHdG), a biomarker for ROS damage [23], and found that the deletion of HuR did not affect its levels in the livers (Figure 1C). In addition, immunostaining for Ki67, a proliferation marker [24], and transmission electron microscopy (TEM) detected a slight decrease without statistical significance in the number of ki67‐positive (Ki67^+ve^) cells and autophagic vacuoles (AVs) in the livers of cKO mice (Figure S2A,B). These data suggest that the deletion of HuR does not appear to affect liver function.

To ensure that livers of cKO mice metabolised APAP normally, we measured the protein levels of hepatic cytochrome P450 2E1 enzyme (CYP2E1), a key enzyme in APAP metabolism, and found that the deletion of HuR did not affect its protein levels (Figure S3A). As expected, the deletion of HuR did not affect CYP2E1 mRNA levels in the livers (Figure S3B). In addition, we measured the levels of hepatic GSH at 0.5 h post‐APAP. Consistent with the results in Figure S3A,B, GSH concentrations were equally reduced in CTR and cKO mice at 0.5 h post‐APAP dosing (Figure S3C). These data suggest that APAP is normally metabolised in the livers of cKO mice.

**FIGURE 2:**
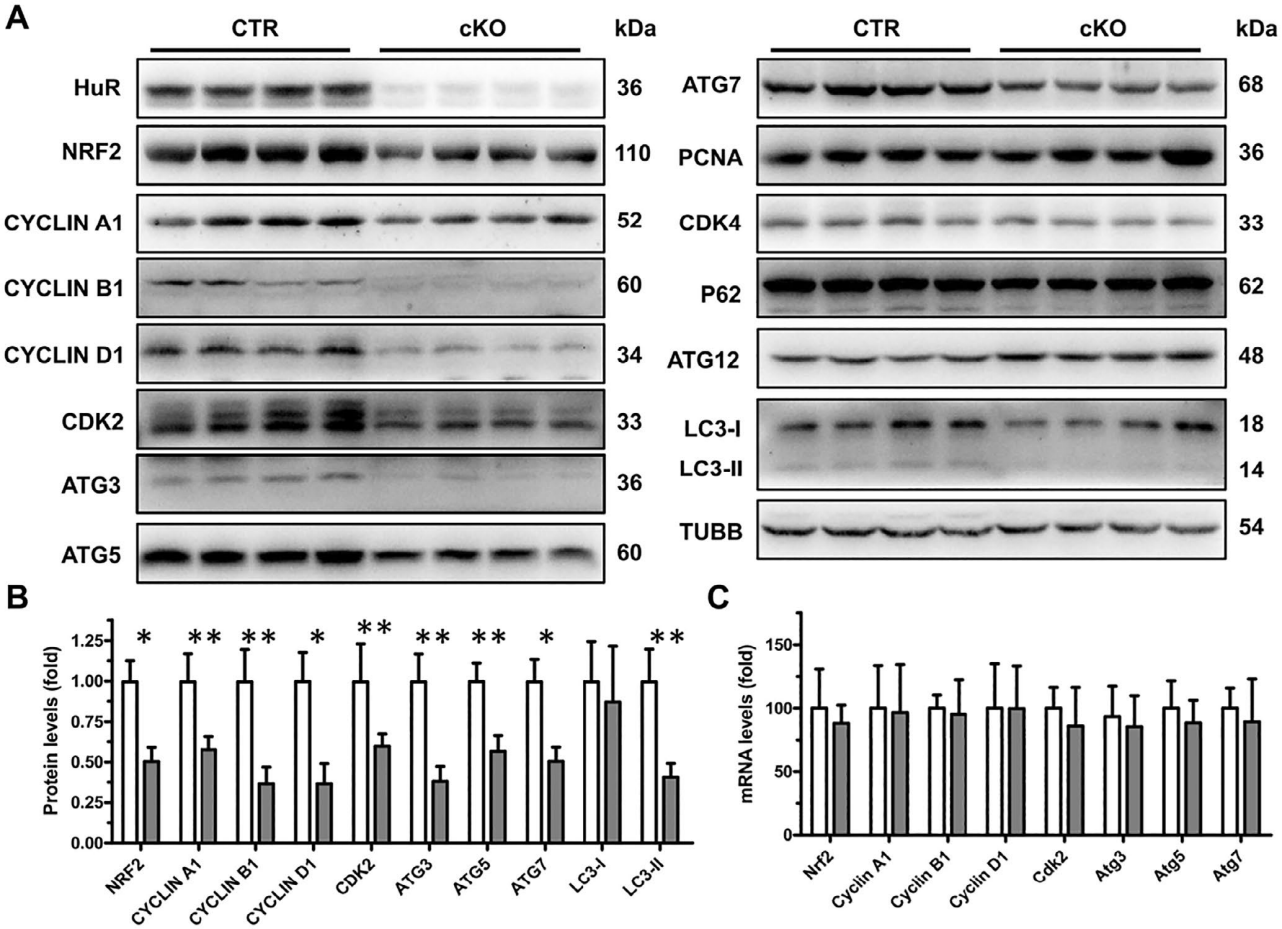
Conditional hepatocyte‐specific HuR knockout results in the reduction of proliferation, autophagy and antioxidant‐related factors. (A) Total liver lysates from CTR and cKO mice were subjected to Western blot analysis for HuR, NRF2, cyclin A1, cyclin B1, cyclin D1, CDK2, ATG3, ATG5, ATG7, PCNA, CDK4, P62, ATG12, LC3‐I, LC3‐II and β‐Tubulin (TUBB). Blots were processed from parallel gels. (B) Densitometry analysis of NRF2, cyclin A1, cyclin B1, cyclin D1, CDK2, ATG3, ATG5, ATG7, LC3‐I and LC3‐II expressions in mice livers (CTR and cKO, *n* = 5). Data are expressed as mean ± SD (blank columns, CTR; black columns, cKO). Significance was determined by a two‐tailed Mann–Whitney U test (*p < 0.05; **p < 0.01). (C) Relative mRNA levels of Nrf2, cyclin A1, cyclin B1, cyclin D1, Cdk2, Atg3, Atg5 and Atg7 in the livers of mice (CTR and cKO, *n* = 5). Data are expressed as mean ± SD (blank columns, CTR; black columns, cKO).

The hepatocyte proliferation, autophagy and antioxidant capacity are essential to ameliorate APAP‐induced liver injury. Therefore, we examined the protein expression of genes related to hepatocyte proliferation and autophagy, as well as NRF2 in the livers of cKO and CTR mice. The levels of NRF2 were reduced by HuR ablation (Figure 2A,B). Similarly, the levels of Nucl‐NRF2 were decreased by HuR ablation (Figure S4A,B). In addition, the levels of cyclin A1, cyclin B1, cyclin D1, CDK2, ATG3, ATG5 and ATG7 decreased significantly in the livers of cKO mice (Figure 2A,B), while the levels of CDK4 and ATG12 in livers were unchanged between cKO and CTR mice (Figure 2A). As expected, the levels of LC3‐II, a molecular marker of autophagy [25], were down‐regulated in the livers of cKO mice (Figure 2A,B). Next, we evaluated hepatocyte proliferation levels in cKO and CTR mice in the absence of APAP treatment. The levels of proliferating cell nuclear antigen (PCNA), a biomarker for the cell proliferation [7], in livers were unchanged between cKO and CTR mice (Figure 2A,B), indicating unchanged hepatocyte proliferation in the livers of cKO mice without APAP treatment, which was consistent with the results in Figure S2A. Although autophagy‐related protein expression was reduced, we wanted to examine whether autophagic flux was impaired in the liver of cKO mice. P62 (also known as SQSTM1/sequestome1) is a specific substrate for autophagy, which is effectively degraded by autophagy [26]; therefore, the stimulation of autophagy flux causes the depletion of p62 and when autophagic clearance is impaired, p62 accumulates within cells [27]. The levels of p62 in livers were unchanged between cKO and CTR mice (Figure 2A,B), indicating that autophagic flux was not impaired in the livers of cKO mice without APAP treatment.

Unlike protein levels, the qPCR results showed that HuR deletion did not affect the levels of Nrf2, cyclin A1, cyclin B1, cyclin D1, Cdk2, Atg3, Atg5 and Atg7 mRNAs (Figure 2C). Consistent with the levels of PCNA, CDK4, P62 and ATG12 (Figure 2A), the levels of PCNA, Cdk4, p62 and Atg12 mRNAs (Figure S4C) also remained unchanged in the livers of cKO mice. Furthermore, we measured the protein and mRNA levels of downstream signalling molecules of NRF2, and the results showed that the protein and mRNA levels of GCLC, GCLM, NQO1, HO‐1 and SOD1 decreased, while the protein and mRNA levels of CAT and SOD2 remained unchanged in the livers of cKO mice (Figure S4D,F). The possible reason might be that NRF2 does not regulate Sod2 and Cat mRNA transcription in mouse hepatocytes. Taken together, these data suggest that HuR regulates hepatocyte proliferation, autophagy and antioxidant capacity.

### 3.2 Conditional Hepatocyte‐Specific HuR Knockout Aggravates APAP‐Induced Liver Injury

**FIGURE 3:**
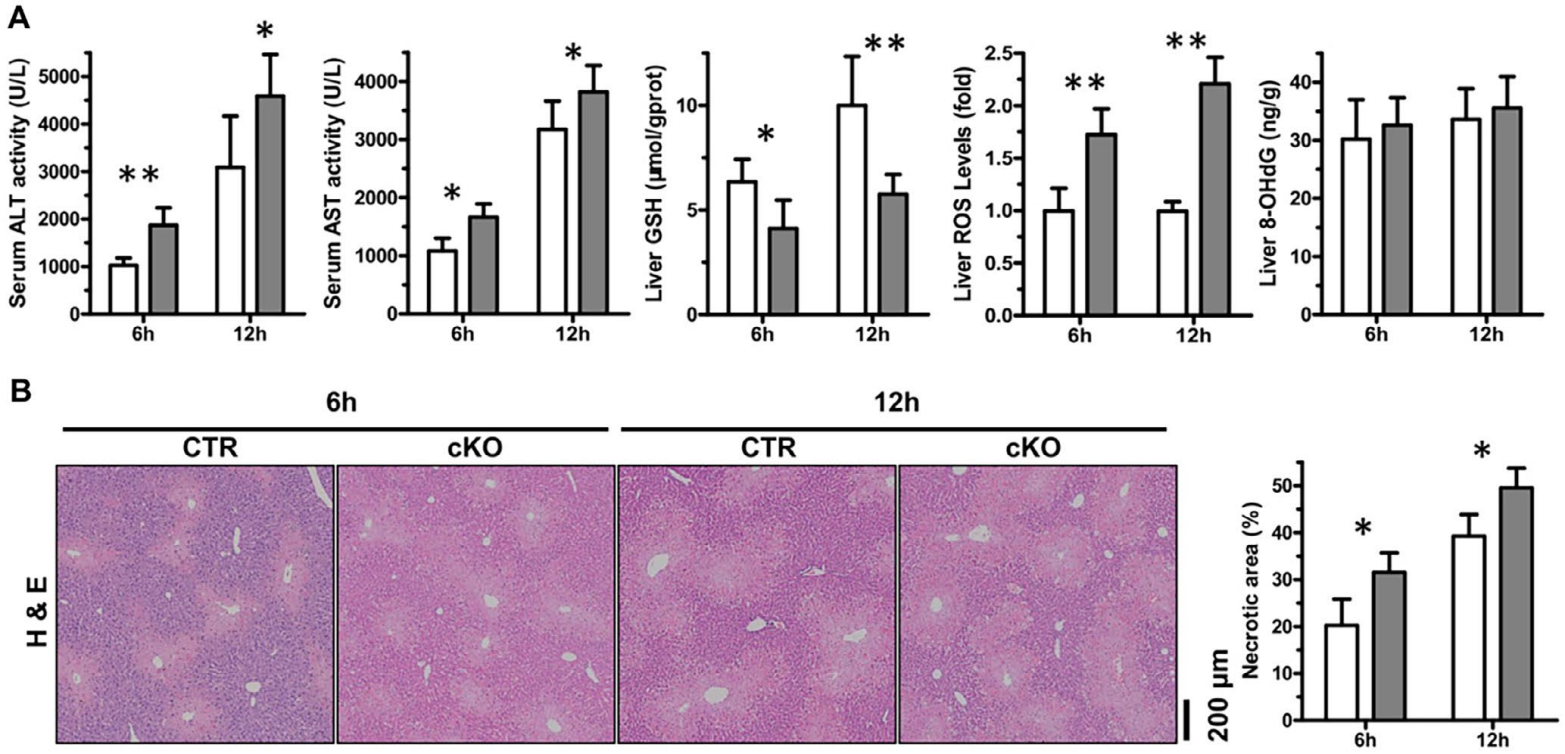
Conditional hepatocyte‐specific HuR knockout aggravates APAP‐induced liver injury. (A) Serum ALT, AST levels, hepatic GSH, ROS and 8‐OHdG levels at 6 and 12 h post‐APAP (CTR and cKO, *n* = 5). Data are expressed as mean ± SD (blank columns, CTR; black columns, cKO). Significance was determined by a two‐tailed Mann–Whitney U test (*p < 0.05; **p < 0.01). (B) Representative H&E‐stained liver photographs at 6 and 12 h post‐APAP and quantification of liver necrotic areas (CTR and cKO, *n* = 4) (scale bars, 200 μm). Data are expressed as mean ± SD (blank columns, CTR; black columns, cKO). Significance was analysed by a two‐tailed Student's *t*‐test (*p < 0.05).

We determined whether the deletion of HuR in hepatocytes was damaging or protective in the context of APAP‐induced liver injury. First, we measured the levels of serum ALT, AST, hepatic GSH, ROS and 8‐OHdG at 6 and 12 h post‐APAP. In the mouse model of APAP‐induced liver injury, the deletion of HuR elevated the serum levels of ALT and AST at 6 and 12 h post‐APAP (Figure 3A). As compared to CTR mice, the levels of hepatic GSH were lower in cKO mice at 6 and 12 h post‐APAP (Figure 3A). Furthermore, the deletion of HuR elevated the levels of hepatic ROS at 6 and 12 h post‐APAP (Figure 3A). Similarly, the levels of hepatic 8‐OHdG showed a slight increase in cKO mice at 6 and 12 h post‐APAP dosing but not statistically significant (Figure 3A). As expected, HuR cKO mice had higher centrilobular necrosis at 6 and 12 h following APAP dosing (Figure 3B). Immunostaining for Ki67 showed a less degree of hepatocyte proliferation in the centrilobular regions of injured cKO livers at 6 and 12 h following APAP dosing, as compared to CTRs (Figure S5A,B). In addition, TEM detected a significant decrease in the number of AVs at 6 and 12 h following APAP dosing in the livers of cKO mice (Figure S6A,B). Taken together, these data show that APAP‐induced liver injury is exaggerated after HuR deletion in the liver.

**FIGURE 4:**
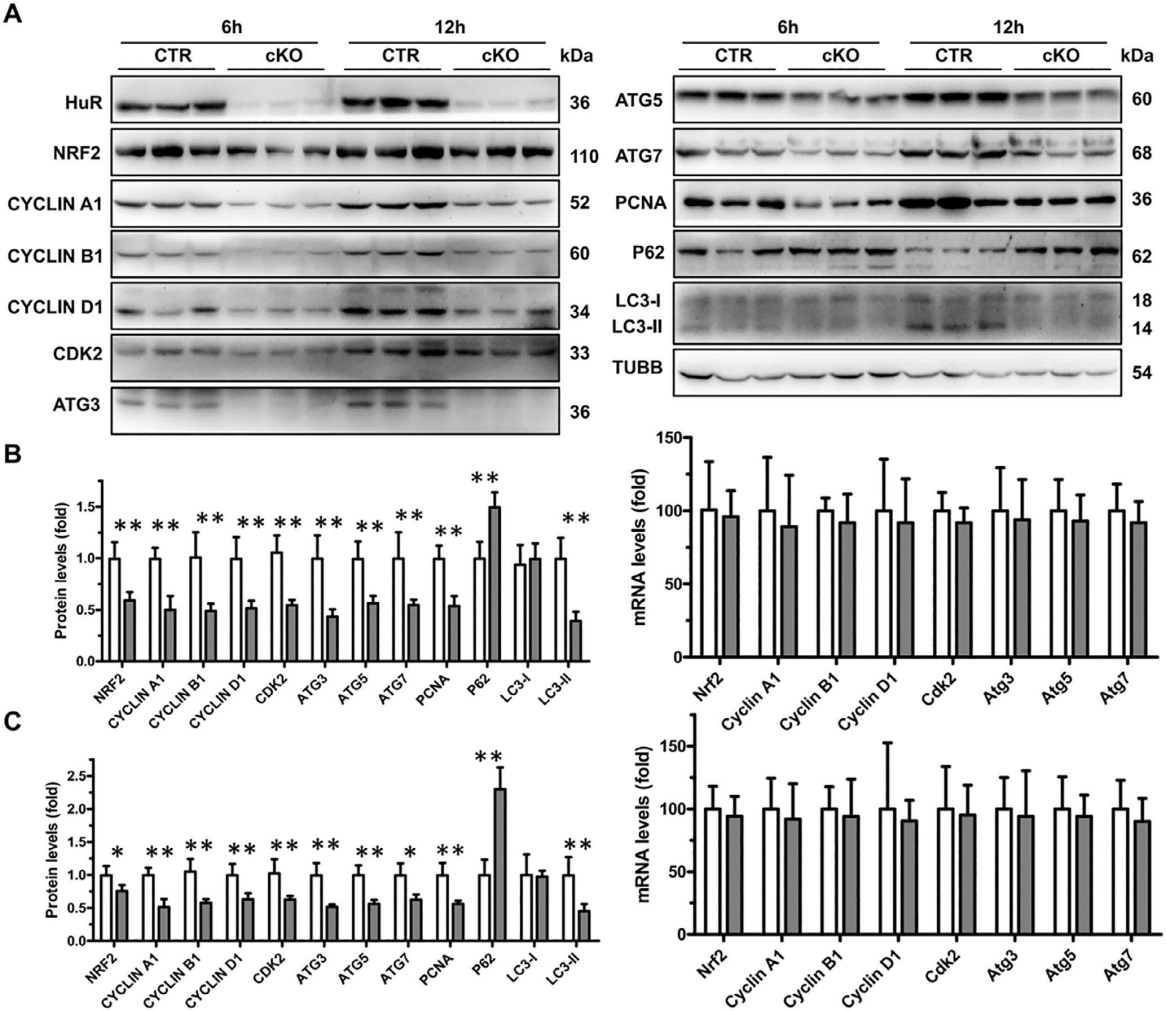
Conditional hepatocyte‐specific HuR knockout leads to the reduction of proliferation, autophagy and antioxidant‐related factors in APAP‐treated mice. (A) CTR and cKO mice were treated with 300 mg/kg of APAP for 6 and 12 h, and total protein lysates from liver tissues were subjected to Western blot analysis for HuR, NRF2, cyclin A1, cyclin B1, cyclin D1, CDK2, ATG3, ATG5, ATG7, PCNA, P62, LC3‐I, LC3‐II and β‐Tubulin (TUBB). Blots were processed from parallel gels. (B) The density of the protein signals for NRF2, cyclin A1, cyclin B1, cyclin D1, CDK2, ATG3, ATG5, ATG7, PCNA, P62, LC3‐I and LC3‐II expressions in mice livers at 6 h post‐APAP dosing (CTR and cKO, *n* = 5), and relative mRNA levels of Nrf2, cyclin A1, cyclin B1, cyclin D1, Cdk2, Atg3, Atg5 and Atg7 in the livers of mice at 6 h post‐APAP dosing (CTR and cKO, *n* = 5). Data are expressed as mean ± SD (blank columns, CTR; black columns, cKO). Significance was determined by a two‐tailed Mann–Whitney U test (**p < 0.01). (C) The density of the signals for NRF2, cyclin A1, cyclin B1, cyclin D1, CDK2, ATG3, ATG5, ATG7, PCNA, P62, LC3‐I and LC3‐II expressions in mice livers at 12 h post‐APAP (CTR and cKO, *n* = 5), and relative mRNA levels of Nrf2, cyclin A1, cyclin B1, cyclin D1, Cdk2, Atg3, Atg5 and Atg7 in the livers from CTR and cKO mice at 12 h post‐APAP (CTR and cKO, *n* = 5). Data are expressed as mean ± SD (blank columns, CTR; black columns, cKO). Significance was determined by a two‐tailed Mann–Whitney U test (*p < 0.05; **p < 0.01).

Unsurprisingly, the levels of NRF2, cyclin A1, cyclin B1, cyclin D1, CDK2, ATG3, ATG5 and ATG7 in the livers of cKO mice were significantly lower than those of CTR mice at 6 and 12 h following APAP dosing (Figure 4A–C). Similarly, the levels of Nucl‐NRF2 in the livers of cKO mice were significantly lower than those of CTR mice at 6 and 12 h post‐APAP (Figure S7A,B). In addition, the levels of LC3‐II were down‐regulated in the livers of cKO mice at 6 and 12 h post‐APAP (Figure 4A–C). Consistent with the results in Figure S5A,B, the levels of PCNA in the livers of cKO mice were significantly lower than those of CTR mice at 6 and 12 h, indicating a decreased hepatocyte proliferation in the livers of cKO mice following APAP dosing (Figure 4A). As expected, the levels of p62 in the livers of cKO mice were significantly higher than those of CTR mice at 6 and 12 h, which indicated that the autophagic degradation was decreased in the livers of cKO mice following APAP dosing (Figure 4A). Consistent with the results in Figure 2C, the levels of Nrf2, cyclin A1, cyclin B1, cyclin D1, Cdk2, Atg3, Atg5 and Atg7 mRNAs in the livers of cKO mice were not affected at 6 and 12 h following APAP dosing (Figure 4B,C). These data show that HuR ameliorates APAP‐induced liver injury by regulating the protein expression of NRF2, cyclin A1, cyclin B1, cyclin D1, CDK2, ATG3, ATG5 and ATG7.

### 3.3 Inhibition of Autophagy Aggravates APAP‐Induced Liver Injury

**FIGURE 5:**
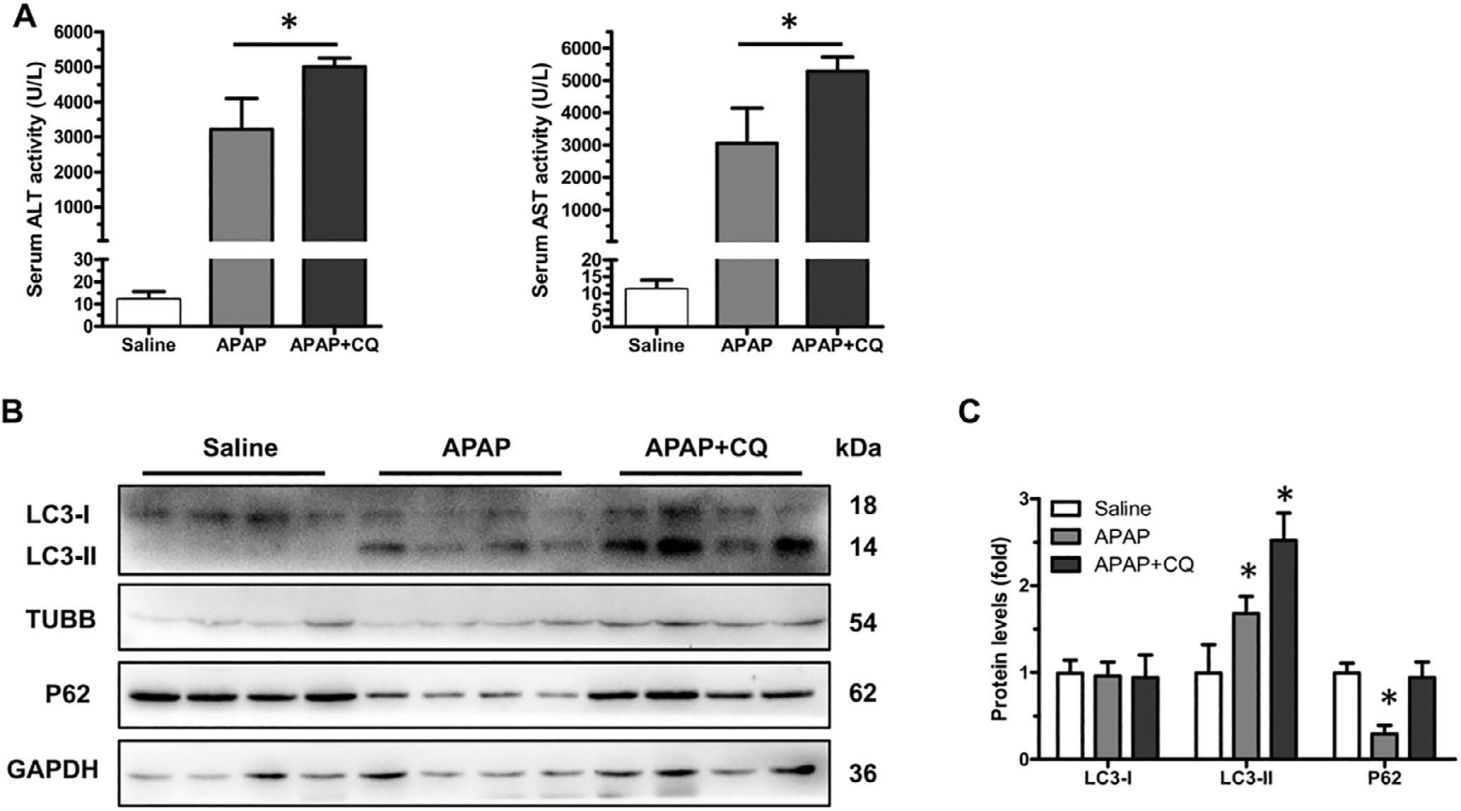
Inhibition of autophagy enhances APAP‐induced liver injury. Male C57BL/6J mice were treated with saline, 300 mg/kg of APAP or 300 mg/kg of APAP +60 mg/kg CQ for 12 h. (A) Serum ALT and AST levels were analysed (saline, *n* = 4; APAP, *n* = 4; APAP + CQ, *n* = 4). Data are expressed as mean ± SD. Significance was determined by a two‐tailed Mann–Whitney U test (*p < 0.05). (B) Total protein lysates from liver tissues were subjected to Western blot analysis for P62, LC3‐I, LC3‐II and β‐Tubulin (TUBB). Blots were processed from parallel gels. (C) Densitometry analysis of P62, LC3‐I and LC3‐II expressions in mice livers (saline, *n* = 4; APAP, *n* = 4; APAP + CQ, *n* = 4). Data are expressed as mean ± SD. Significance was determined by a two‐tailed Mann–Whitney U test (*p < 0.05).

To further confirm that autophagy is a protective mechanism limiting APAP toxicity, we next measured the levels of ALT and AST when mice were treated with APAP in the presence or absence of CQ, which inhibits autophagic degradation by preventing the fusion of autophagosomes and lysosomes. We found that serum ALT and AST levels were significantly higher in APAP and CQ‐treated mice compared to those in APAP alone‐treated mice (Figure 5A), suggesting that the inhibition of autophagy by CQ further exacerbated APAP‐induced liver injury. As expected, the treatment of mice with CQ further increased the levels of LC3‐II and restored the levels of p62 (Figure 5B,C). Therefore, these findings suggest that autophagy played an active role in limiting APAP‐induced liver injury.

### 3.4 HuR and Its Downstream Target Expression Are Up‐Regulated in APAP‐Induced Liver Injury

**FIGURE 6:**
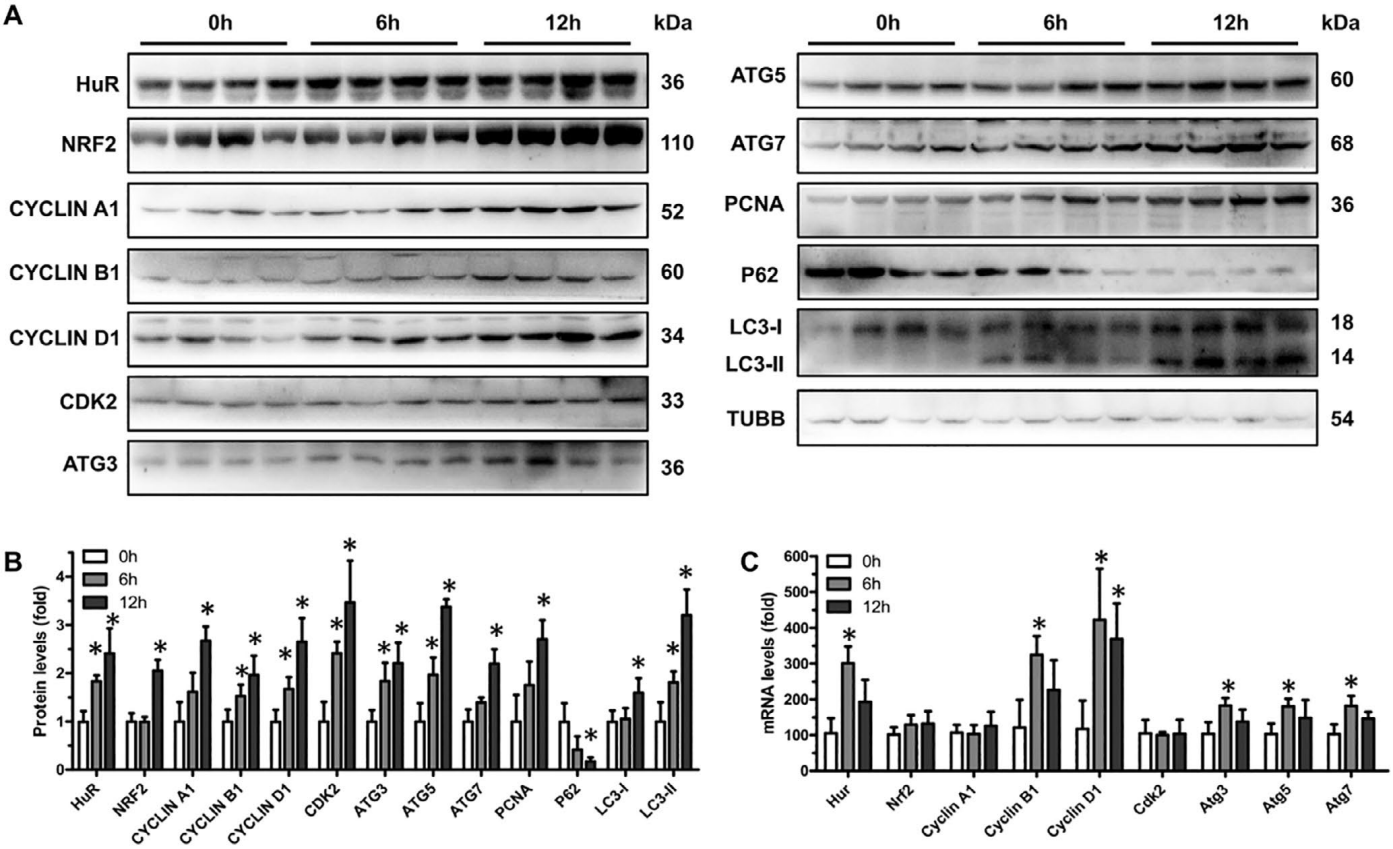
The protein expression of HuR, NRF2, cyclin A1, cyclin B1, cyclin D1, CDK2, ATG3, ATG5, ATG7, PCNA, P62, LC3‐I and LC3‐II are increased in the livers of mice treated with APAP. (A) Protein lysates from liver tissues described in Figure S8A were subjected to Western blot analysis for HuR, NRF2, cyclin A1, cyclin B1, cyclin D1, CDK2, ATG3, ATG5, ATG7, PCNA, P62, LC3‐I, LC3‐II and β‐Tubulin (TUBB). Blots were processed from parallel gels. (B) Densitometry analysis of HuR, NRF2, cyclin A1, cyclin B1, cyclin D1, CDK2, ATG3, ATG5, ATG7, PCNA, P62, LC3‐I and LC3‐II expressions in mice livers at 0, 6 and 12 h post‐APAP dosing (0 h, *n* = 4; 6 h, *n* = 4; 12 h, *n* = 4). Data are expressed as mean ± SD. Significance was determined by a two‐tailed Mann–Whitney U test (*p < 0.05). (C) Relative mRNA levels of Hur, Nrf2, cyclin A1, cyclin B1, cyclin D1, Cdk2, Atg3, Atg5 and Atg7 in the livers of mice at 0, 6 and 12 h post‐APAP dosing (0 h, *n* = 4; 6 h, *n* = 4; 12 h, *n* = 4). Data are expressed as mean ± SD. Significance was determined by a two‐tailed Mann–Whitney U test (*p < 0.05).

To investigate the protein expression of HuR and its downstream targets in APAP‐induced liver injury, we made a model of liver injury in C57BL/6J mice. After confirming liver injury in mice by serum and liver biochemistry analysis and H&E staining (Figure S8A,B), we examined the protein expression levels of HuR and its downstream targets in the livers. As shown in Figure 6A,B, the protein levels of HuR, NRF2, cyclin A1, cyclin B1, cyclin D1, CDK2, ATG3, ATG5 and ATG7 continued to increase at 6 and 12 h post‐APAP dosing. Unsurprisingly, the protein levels of Nucl‐NRF2 continued to increase at 6 and 12 h post‐APAP dosing (Figure S8C,D). Similarly, the protein levels of LC3‐II continued to increase at 6 and 12 h post‐APAP dosing (Figure 6A,B), indicating enhanced autophagy after liver injury. As expected, the protein levels of PCNA continued to increase at 6 and 12 h post‐APAP dosing, while p62 protein levels continued to decrease (Figure 6A,B). The levels of HuR mRNA were an increase in the livers at 6 and 12 h post‐APAP dosing (Figure 6C). Surprisingly, C57BL/6J mice treating with APAP exhibited increased levels of cyclin B1, cyclin D1, Atg3, Atg5 and Atg7 mRNAs but not Nrf2, cyclin A1, cyclin D1 or Cdk2 mRNAs (Figure 6C). The reason for the mRNA elevation may be that these genes are regulated by other factors besides HuR. We therefore propose that APAP can induce liver injury while also increasing the levels of HuR, which play a role in improving liver injury.

### 3.5 HuR Promotes mRNA Translation by Binding to the 3′UTRs of Its Downstream Target mRNAs

**FIGURE 7:**
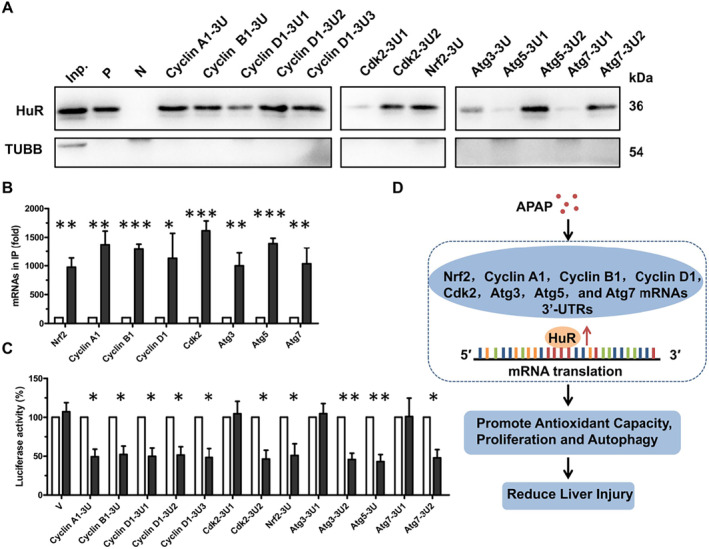
The mechanisms that HuR up‐regulating the expression of NRF2, cyclin A1, cyclin B1, cyclin D1, CDK2, ATG3, ATG5 and ATG7. (A) RNA pull‐down analysis was conducted by using Hepa1‐6 cell lysates and in vitro‐transcribed RNAs described in Figure S9. Ndufb6 5′UTR and Ndufb6 3′UTRs were used as positive control (P) and negative control (N), respectively. The input (Inp.) and TUBB were also evaluated. (B) RNA immunoprecipitation with anti‐HuR antibody or control IgG was conducted by using Hepa1‐6 cell lysates. Data are expressed as mean ± SD (blank columns, IgG; black columns, HuR). Significance was analysed by a two‐tailed Student's *t*‐test (*p < 0.05; **p < 0.01; *p < 0.001). (C) Hepa1‐6 cells were transfected with control or HuR‐directed siRNAs for 48 h, and then continued to be transfected with reporter plasmids depicted in Figure S10 for 24 h, whereupon the relative luciferase activities were measured. PGL3‐promoter vector (V) was used as a control. Data are expressed as mean ± SD (blank columns, control; black columns, siHuR). Significance was analysed by a two‐tailed Student's *t*‐test (*p < 0.05; **p < 0.01). (D) A sketch figure for possible roles of HuR ameliorating APAP‐induced liver injury by binding to the 3′UTRs of Nrf2, cyclin A1, cyclin B1, cyclin D1, Cdk2, Atg3, Atg5 and Atg7 mRNAs and promoting their translation.

To further investigate the regulatory mechanisms of HuR on its downstream targets, we performed in vitro RNA pull‐down assays using biotin‐labelled RNA fragments of the 3′UTRs of cyclin A1, cyclin B1, cyclin D1, Cdk2, Nrf2, Atg3, Atg5 and Atg7 mRNAs (Figure S9A–H, Schematic), followed by Western blotting with HuR and TUBB antibody. Figure 7A shows that HuR associated with the fragments of cyclin A1‐3U, cyclin B1‐3U, cyclin D1‐3U1, cyclin D1‐3U2, cyclin D1‐3U3, Cdk2‐3U2, Nrf2‐3U, Atg3‐3U, Atg5‐3U2 and Atg7‐3U2 but not with other regions of these mRNAs. In addition, an RNA immunoprecipitation (RIP) assay showed that HuR could bind to Nrf2, cyclin A1, cyclin B1, cyclin D1, Cdk2, Atg3, Atg5 and Atg7 mRNAs (Figure 7B). To further research HuR‐mediated regulation of Nrf2, cyclin A1, cyclin B1, cyclin D1, Cdk2, Atg3, Atg5 and Atg7 mRNAs, we performed double‐luciferase reporter assays. PGL3‐derived reporter plasmids were constructed bearing the 3′UTRs of Nrf2, cyclin A1, cyclin B1, cyclin D1, Cdk2, Atg3, Atg5 and Atg7 mRNAs, as shown in Figure S10. The results showed that lowering HuR resulted in a reduction of the firefly luciferase activity/Renilla luciferase activity ratios in pGL3‐derived reporter plasmids bearing cyclin A1‐3U, cyclin B1‐3U, cyclin D1‐3U1, cyclin D1‐3U2, cyclin D1‐3U3, Cdk2‐3U2, Nrf2‐3U, Atg3‐3U2, Atg5‐3U and Atg7‐3U2 but not with other reporter plasmids bearing fragments that did not combine with HuR (Figure 7C). These data suggest that HuR associated with the 3′UTRs of Nrf2, cyclin A1, cyclin B1, cyclin D1, Cdk2, Atg3, Atg5 and Atg7 mRNAs and promoted their translation.

## 4 Discussion

As APAP overdose is still common, DILI has become a major health burden for countries. Unfortunately, treatment options are limited. Activation of HuR has been reported to attenuate non‐alcoholic fatty liver disease [21] and liver inflammation and fibrosis [28], but its role in DILI has not been reported.

In this study, we investigated the effect of conditional hepatocyte‐specific HuR knockout on APAP‐induced liver injury. Our data showed that APAP induced HuR expression in mice livers, which prevented APAP‐induced liver injury by regulating hepatocyte proliferation, autophagy and antioxidant capacity (Figure 7D). Mechanistically, HuR could associate with the 3′UTRs of Nrf2, cyclin A1, cyclin B1, cyclin D1, Cdk2, Atg3, Atg5 and Atg7 mRNAs and thereby promote the expression of NRF2, cyclin A1, cyclin B1, cyclin D1, CDK2, ATG3, ATG5 and ATG7. Taken together, our data suggest that HuR may play an important role in APAP‐induced liver injury.

HuR, a post‐transcriptional regulator, mediates RNA splicing and translation by binding to AU‐rich elements (AREs), typically present in the 3′UTRs of its target transcripts [29]. A recent research reported that HuR bound to Sod2 mRNA and enhanced its translation under anchorage‐independence in OVCA433 and OVCAR10 cells [30]. In addition, HuR controls liver lipid homeostasis by associating with the 3′UTRs of Cycs, Ndufb6 and Uqcrb mRNAs, as well as Apob pre‐mRNA [21]. In this study, we found that the HuR protein expression was inducible in the mouse model of APAP‐induced liver injury and found that HuR could protect against APAP‐induced liver injury by promoting the expression of NRF2, cyclin A1, cyclin B1, cyclin D1, CDK2, ATG3, ATG5 and ATG7. This study not only provides a new hepatic signalling pathway for drug therapy of APAP‐induced liver injury but also complements the biological functions of HuR.

Hepatocyte proliferation is critical for liver repair and decides the ultimate result of APAP‐induced liver injury [31]. In agreement with the previous study [18], we also found that HuR regulated cyclin A1 and cyclin B1 expressions in mouse hepatocytes. According to reports, HuR interacted with CircCCNB1 to increase the expression of cyclin D1 in glioma cells [32]. In this context, HuR in hepatocytes could bind to the 3′UTR of cyclin D1 mRNA and promote its expression. A recent study reported that VPS9D1‐AS1 interacted with HuR to affect the stability and expression of the CDK4 mRNA, thus impacting hepatocellular carcinoma (HCC) cell proliferation [33]. Surprisingly, in our study, we found that HuR could bind to CDK2 mRNA and promote its expression in mouse hepatocytes but not CDK4. These results suggest that the pathway by which HuR influences cell proliferation may be different in different cells.

Autophagy is another essential factor in protecting against APAP‐induced liver injury [4]. According to reports, HuR promoted Atg7 mRNA stability by binding to the AU‐rich elements in the 3′UTR in nucleus pulposus (NP) cells [34]. Unlike the mechanisms described above, HuR mediated the translation of ATG5 and ATG12 mRNAs through binding to their 3′UTRs in human liver cells [35]. Here, we found that HuR could bind to the 3′UTRs of Atg3, Atg5 and Atg7 mRNAs but not Atg12 mRNA and promote their translation, which improves our comprehension of the regulatory mechanisms of autophagy in mouse hepatocytes.

Both the APAP‐ADs formed by the binding of NAPQI to intracellular proteins and the depletion of GSH could cause oxidative stress and ultimately exert hepatotoxicity effects; therefore, oxidative stress is considered one of the key mechanisms involved in APAP‐induced liver injury and a potential therapeutic target [36]. NRF2 represents an essential regulator of the cellular defence mechanisms against oxidative stress. It was reported that HuR could bind to the 3′UTR of Nrf2 mRNA and promote its maturation and nuclear export in HEK293T cells [20]. Thus, we tried to link HuR with the NRF2 expression in mouse hepatocytes. In this context, we found that HuR associated with the 3′UTR of Nrf2 mRNA and regulated its translation, thereby promoting its protein expression. As mentioned earlier, NRF2 regulates several antioxidant enzymes and the rate‐limiting enzymes of GSH synthesis. Thus, in the mouse model of APAP‐induced liver injury, the deletion of HuR led to the decrease of the expression of its downstream targets, which elevated the levels of hepatic ROS and degraded the levels of hepatic GSH. Contrary to our expectations, 8‐OHdG, a biomarker of DNA oxidative damage, was not significantly elevated. The possible reason might be that the time for APAP treatment of mice was too short to cause much change.

In conclusion, we showed that HuR functions as an indispensable regulator of APAP‐induced liver injury by controlling the production of NRF2, cyclin A1, cyclin B1, cyclin D1, CDK2, ATG3, ATG5 and ATG7. Conditional hepatocyte‐specific knockout of HuR aggravated APAP‐induced liver injury, which suggested that HuR could serve as a potential therapeutic target for APAP‐induced liver injury.

## Author Contributions

**Chen Zong:** conceptualization (lead), funding acquisition (lead), project administration (lead), supervision (lead), visualization (equal), writing – original draft (equal). **Linlin Lu:** investigation (lead), methodology (lead), validation (lead). **Jicui Chen:** formal analysis (lead), funding acquisition (equal), resources (lead). **Hui Jiang:** investigation (equal), validation (equal). **Ni Li:** formal analysis (equal). **Xiaojie Li:** project administration (equal). **Yinghao Liu:** writing – original draft (lead).

## Conflicts of Interest

The authors declare no conflicts of interest.

## Supporting information


Data S1.

## Data Availability

All data are available upon request.
