# Supplementary material for: Hepatocyte‐Specific HuR Protects Against Acetaminophen‐Induced Liver Injury in Mice
Source: J Cell Mol Med. 2024 Nov 29;28(22):e70246. doi: 10.1111/jcmm.70246 (PMC11606931; doi:10.1111/jcmm.70246)
Supplement: Supplementary file 1 — Data S1. [file JCMM-28-e70246-s001.docx]

**
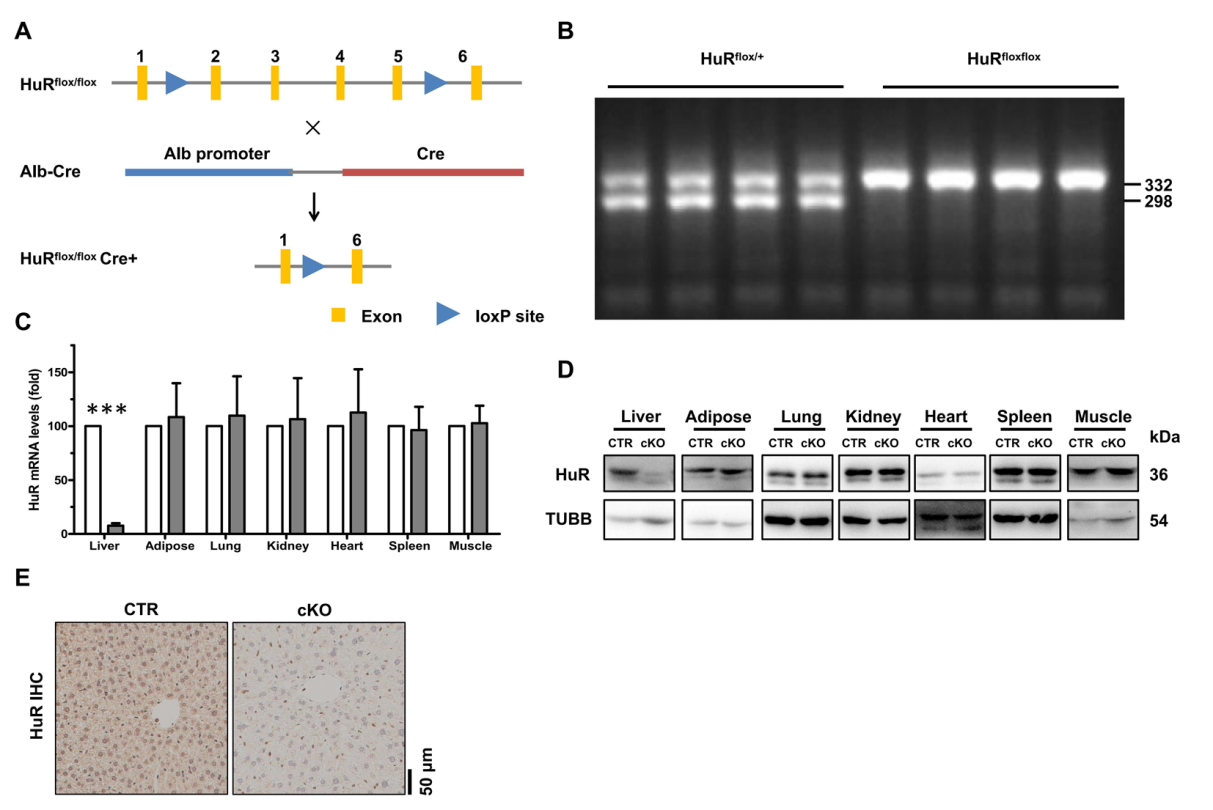
**

**Supplementary Fig. 1.** Generation of conditional hepatocyte-specific HuR knockout mice. (A) Schematic of transgenic mice used to generate cKO mice. (B) Representative genotyping of HuR^flox/+^ and HuR^flox/flox^ mice. (C) Relative mRNA levels of HuR in tissues from CTR and cKO mice (one mouse tissues). Data are expressed as mean ± SD (Blank columns, CTR; Black columns, cKO). Significance was analyzed by two-tailed Student’s t-test (*p < 0.001). (D) Western blot analysis was used to evaluate HuR protein levels in tissues from CTR and cKO mice. (E) Representative immunohistochemistry (IHC) staining images of HuR expression in livers from CTR and cKO mice. (scale bars, 50 µm).


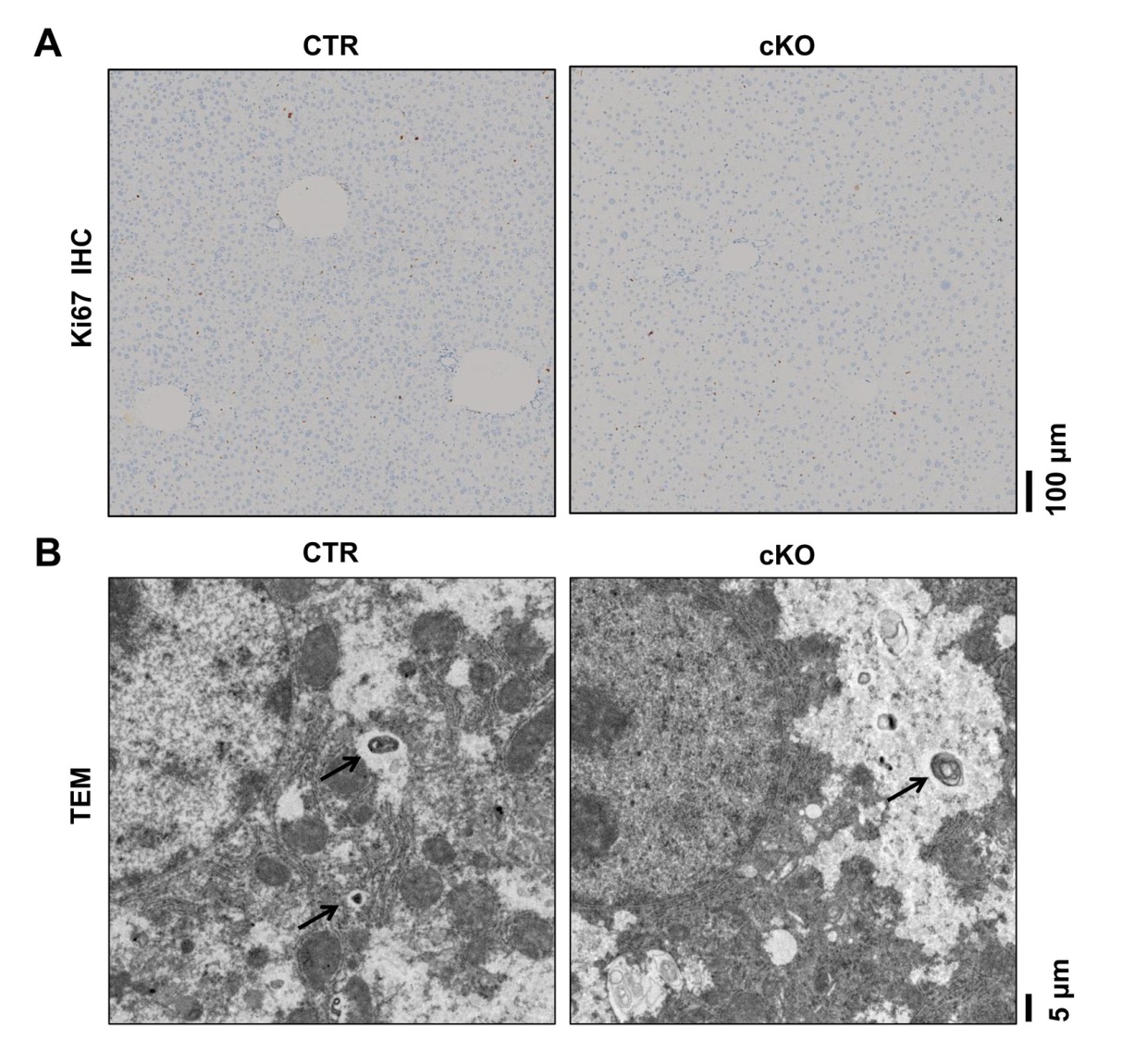


**Supplementary Fig. 2.** Influence of conditional hepatocyte-specific HuR knockout on the number of Ki67 positive cells and autophagic vacuoles. (A) Representative IHC staining images of Ki67 in the livers from CTR and cKO mice (scale bars, 100 µm). (B) Representative liver electron micrographs (scale bars, 5 µm). The arrowheads indicate the autophagosomes and autolysosomes.


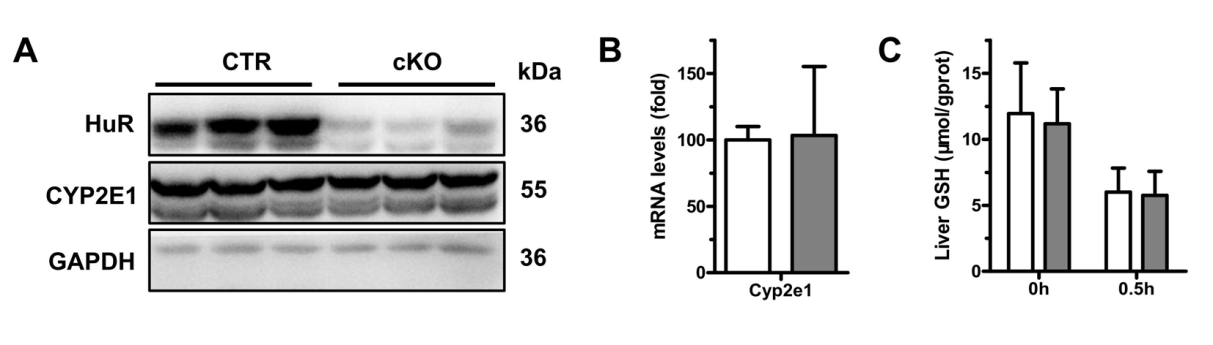


**Supplementary Fig. 3.** Hepatic CYP2E1 levels in mice with conditional hepatocyte-specific HuR knockout and control mice. (A) Total liver lysates from CTR and cKO mice were subjected to western blot analysis to evaluate the levels of HuR, CYP2E1, and GAPDH. Blots were processed from parallel gels. (B) Relative mRNA levels of Cyp2e1 in the livers of mice (CTR and cKO, n = 5). Data are expressed as mean ± SD (Blank columns, CTR; Black columns, cKO). (C) Hepatic GSH levels at 0 and 0.5 h post-APAP (CTR and cKO, n = 5). Data are expressed as mean ± SD (Blank columns, CTR; Black columns, cKO).


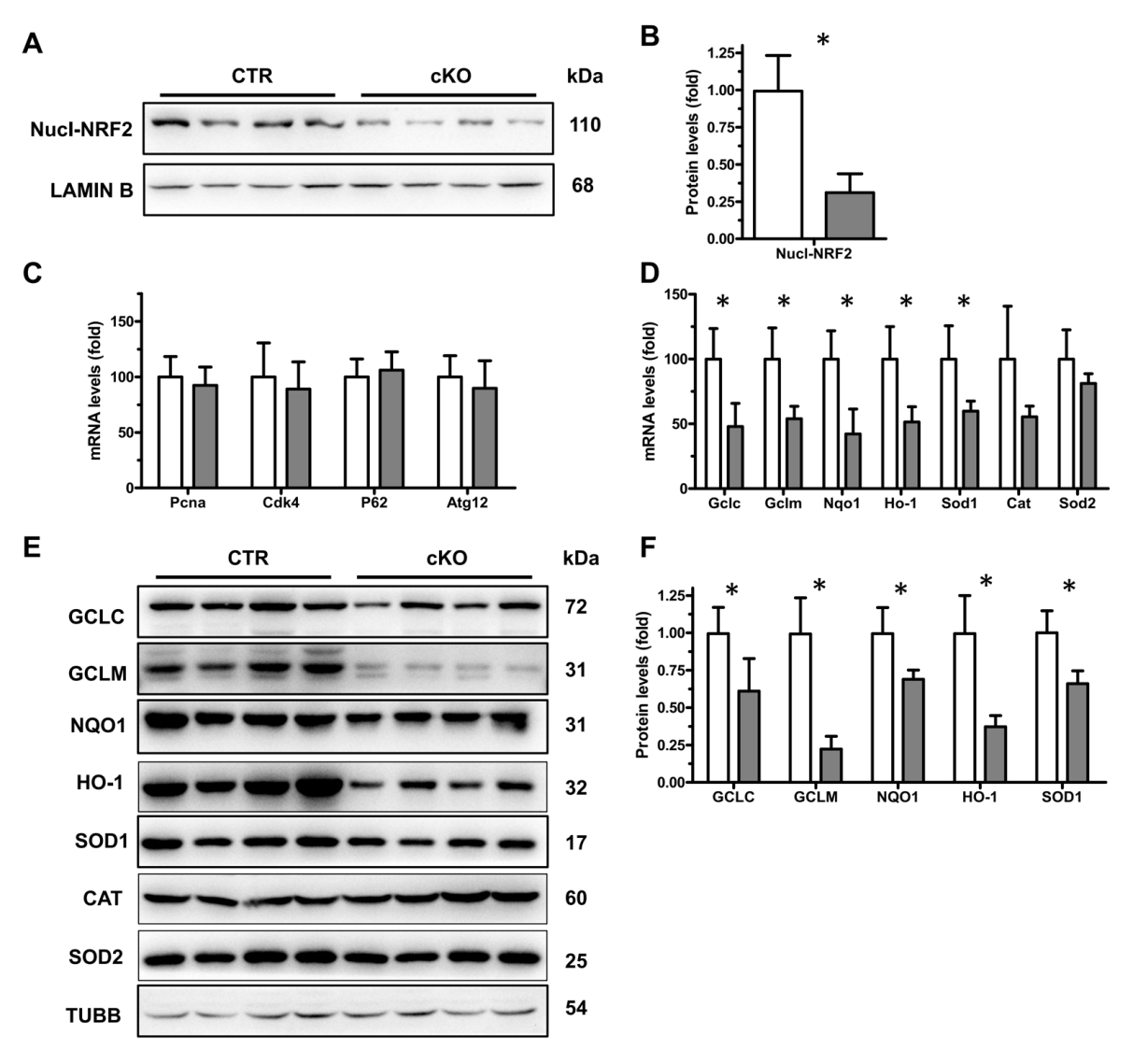


**Supplementary Fig. 4.** Influence of conditional hepatocyte-specific HuR knockout on the protein or mRNA levels of proliferation, autophagy and antioxidant-related factors. (A) The nuclear protein lysates from CTR and cKO mice liver tissues were subjected to western blot analysis for Nucl-NRF2 and LAMIN B. Blots were processed from parallel gels. (B) Densitometry analysis of Nucl-NRF2 expression in mice livers (CTR and cKO, n = 4). Data are expressed as mean ± SD (Blank columns, CTR; Black columns, cKO). Significance was determined by two-tailed Mann-Whitney U test (*p < 0.05). (C) Relative mRNA levels of Pcna, Cdk4, P62, and Atg12 in the livers of mice (CTR and cKO, n = 5). Data are expressed as mean ± SD (Blank columns, CTR; Black columns, cKO). (D) Relative mRNA levels of Gclc, Gclm, Nqo1, Ho-1，Sod1, Cat and Sod2 in the livers of mice (CTR and cKO, n = 4). Data are expressed as mean ± SD (Blank columns, CTR; Black columns, cKO). Significance was determined by two-tailed Mann-Whitney U test (*p < 0.05). (E) Total liver lysates from CTR and cKO mice were subjected to western blot analysis for GCLC, GCLM, NQO1, HO-1, SOD1, CAT, SOD2, and β-Tubulin (TUBB). Blots were processed from parallel gels. (F) Densitometry analysis of GCLC, GCLM, NQO1, HO-1, and SOD1 expression in mice livers (CTR and cKO, n = 4). Data are expressed as mean ± SD (Blank columns, CTR; Black columns, cKO). Significance was determined by two-tailed Mann-Whitney U test (*p < 0.05).


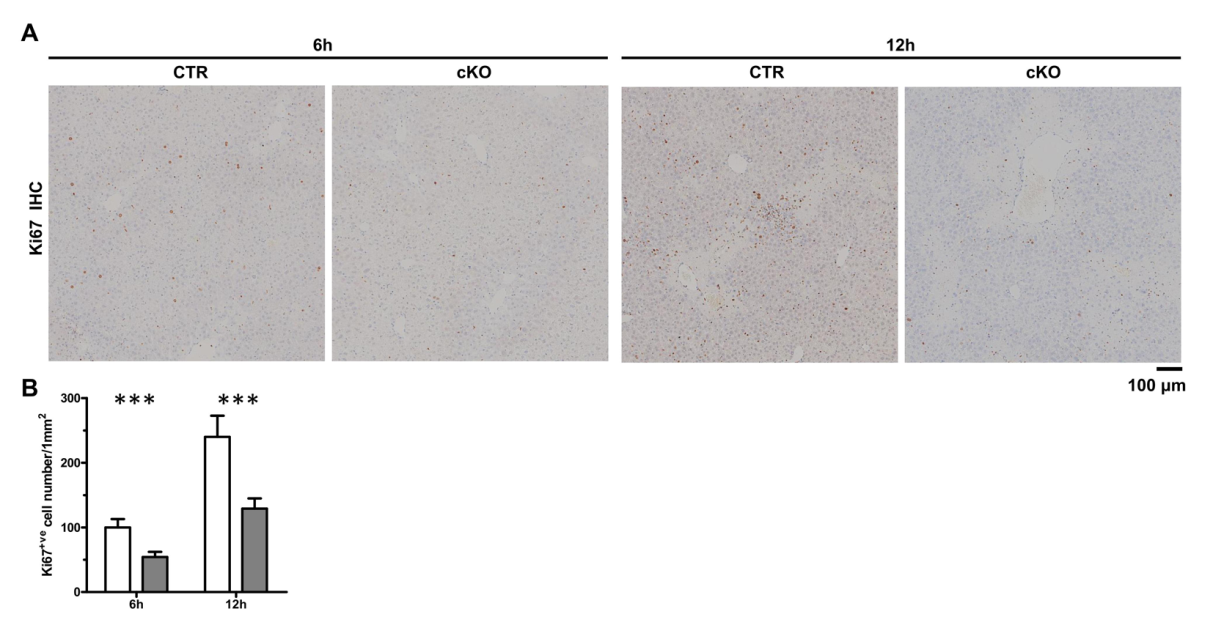


**Supplementary Fig. 5.** Influence of conditional hepatocyte-specific HuR knockout on the number of Ki67 positive cells in APAP-treated mice. (A) CTR and cKO mice were treated with 300 mg/kg APAP for 6 and 12 h, and representative IHC staining images of Ki67 in the livers from the mice (CTR and cKO, n = 5) (scale bars, 100 µm). (B) Quantification of Ki67^+ve^ cells for experiments shown in (A). Data are expressed as mean ± SD (Blank columns, CTR; Black columns, cKO). Significance was analyzed by two-tailed Student’s t-test (***p < 0.001).


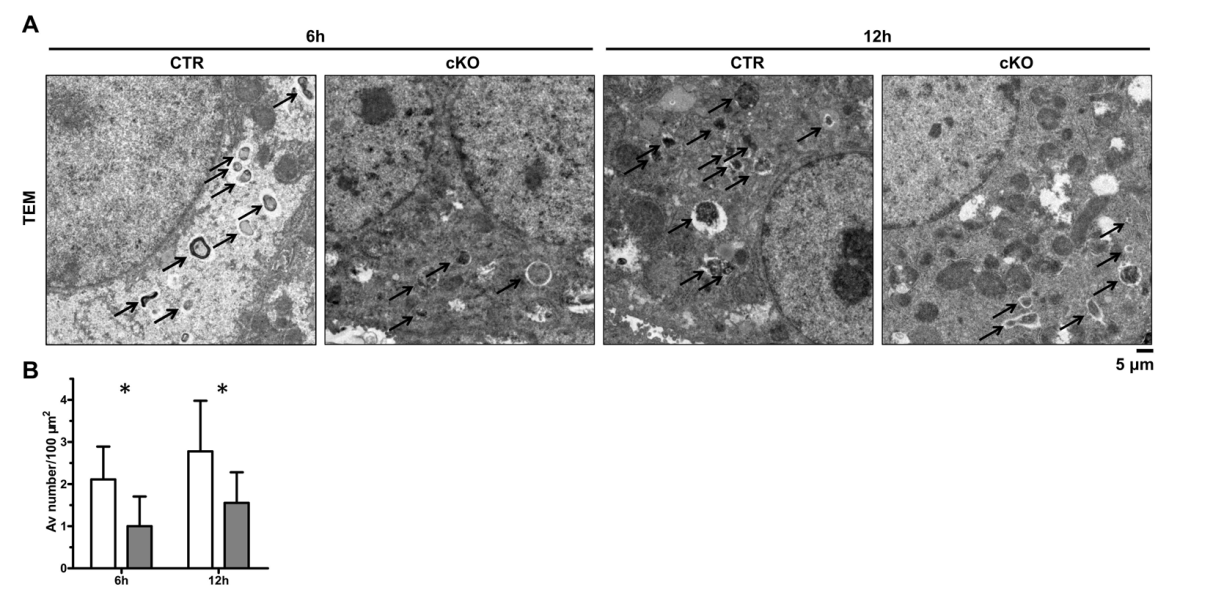


**Supplementary Fig. 6.** Influence of conditional hepatocyte-specific HuR knockout on the number of autophagic vacuoles in APAP-treated mice. (A) Representative liver electron micrographs from the mice described in supplementary Fig. 5A (scale bars, 5 µm). The arrowheads indicate the autophagosomes and autolysosomes. (B) Quantification of autophagic vacuoles (AVs) for experiments shown in (A). Data are expressed as mean ± SD (Blank columns, CTR; Black columns, cKO). Significance was analyzed by two-tailed Student’s t-test (*p < 0.05).


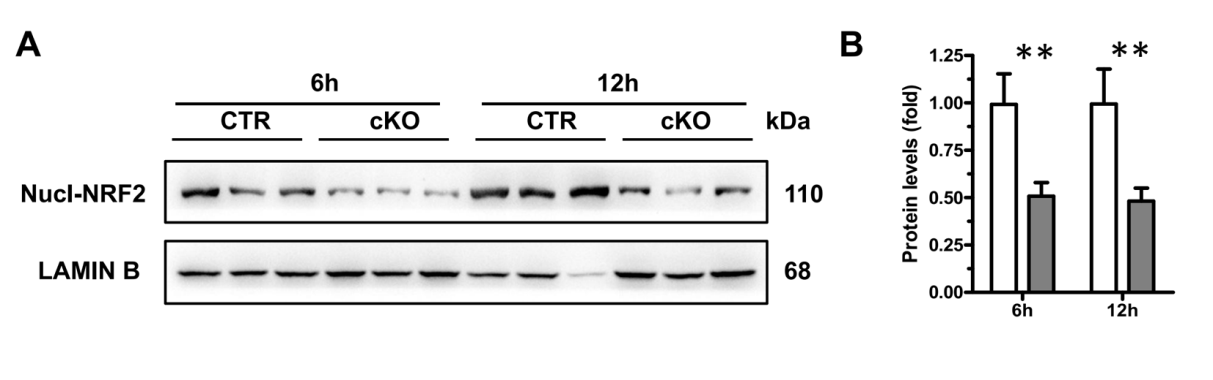


**Supplementary Fig. 7.** Conditional hepatocyte-specific HuR knockout leads to the reduction of Nucl-NRF2 in APAP-treated mice. (A) CTR and cKO mice were treated with 300 mg/kg APAP for 6 and 12 h, and the nuclear protein lysates from liver tissues were subjected to western blot analysis for Nucl-NRF2 and LAMIN B. Blots were processed from parallel gels. (B) The density of the protein signals for Nucl-NRF2 expression in mice livers at 6 h and 12 h post-APAP doing (CTR and cKO, n = 5). Data are expressed as mean ± SD (Blank columns, CTR; Black columns, cKO). Significance was determined by two-tailed Mann-Whitney U test (**p < 0.01).


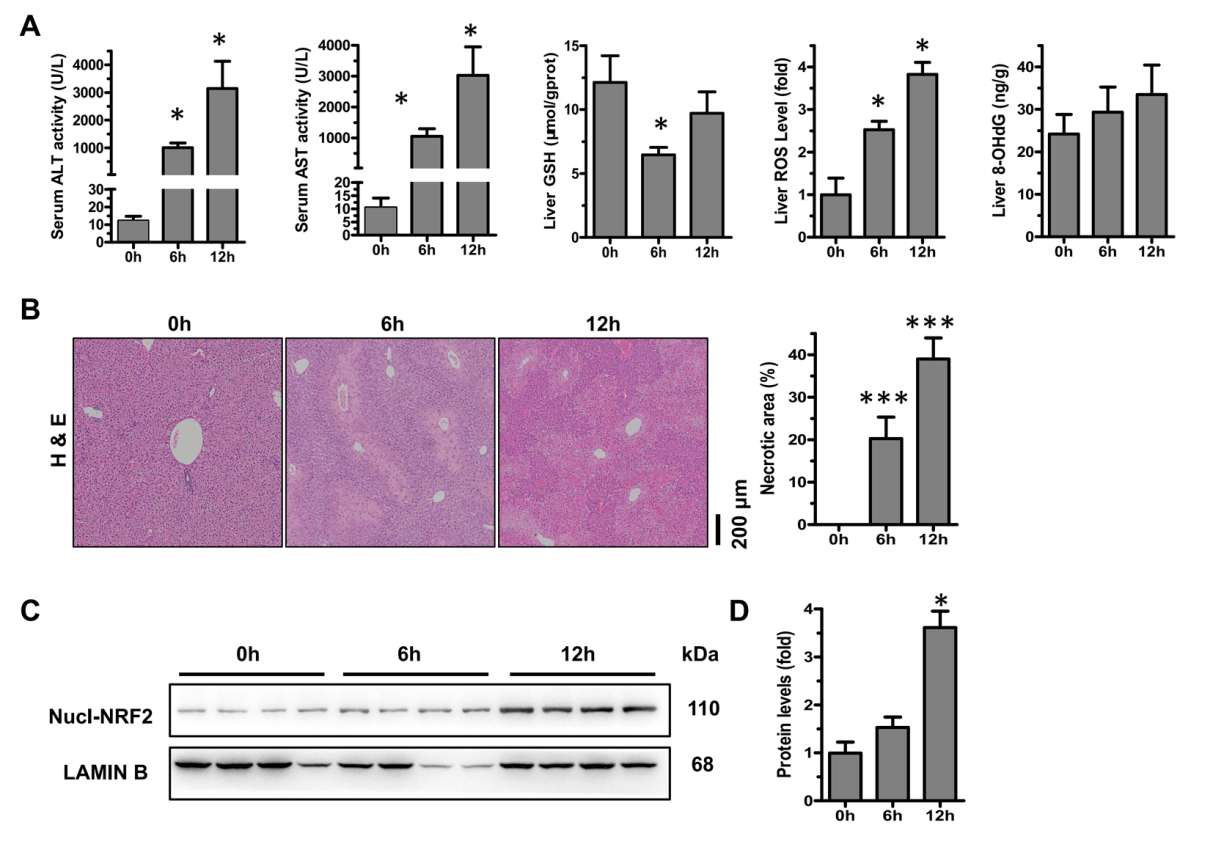


**Supplementary Fig. 8.** 300 mg/kg APAP induces liver injury and increases the expression of Nucl-NRF2 in the livers of mice. (A) Male C57BL/6J mice were treated with 300 mg/kg APAP for 0, 6 and 12 h, and serum ALT, AST levels, hepatic GSH, ROS, and 8-OHdG levels were analyzed (0 h, n = 4; 6 h, n = 4; 12 h, n = 4). Data are expressed as mean ± SD. Significance was determined by two-tailed Mann-Whitney U test (*p < 0.05). (B) Representative H&E-stained liver images at 0, 6 and 12 h post-APAP doing and quantification of liver necrotic areas (0 h, n = 4; 6 h, n = 4; 12 h, n = 4) (scale bars, 100 µm). Data are expressed as mean ± SD. Significance was analyzed by two-tailed Student’s t-test (***p < 0.001). (C) The nuclear protein lysates from liver tissues described in (A) were subjected to western blot analysis for Nucl-NRF2 and LAMIN B. Blots were processed from parallel gels. (D) Densitometry analysis of Nucl-NRF2 expression in mice livers at 0, 6 and 12 h post-APAP doing (0 h, n = 4; 6 h, n = 4; 12 h, n = 4). Data are expressed as mean ± SD. Significance was determined by two-tailed Mann-Whitney U test (*p < 0.05).

**
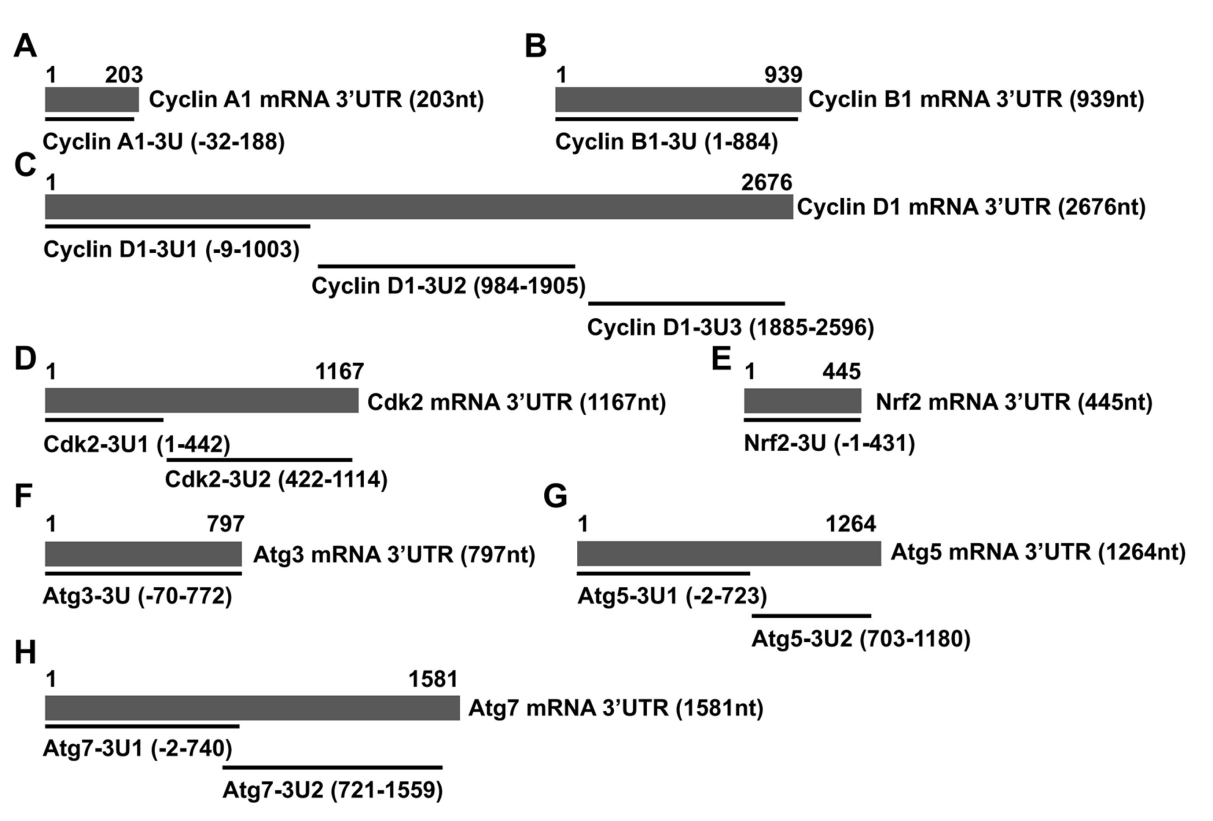
**

**Supplementary Fig. 9.** (A-H) Schematics representation of the CYCLIN A1, CYCLIN B1, CYCLIN D1, CDK2, NRF2, ATG3, ATG5 and ATG7 3′UTRs fragments used for RNA pulldown assays. The location of the fragments is indicated.


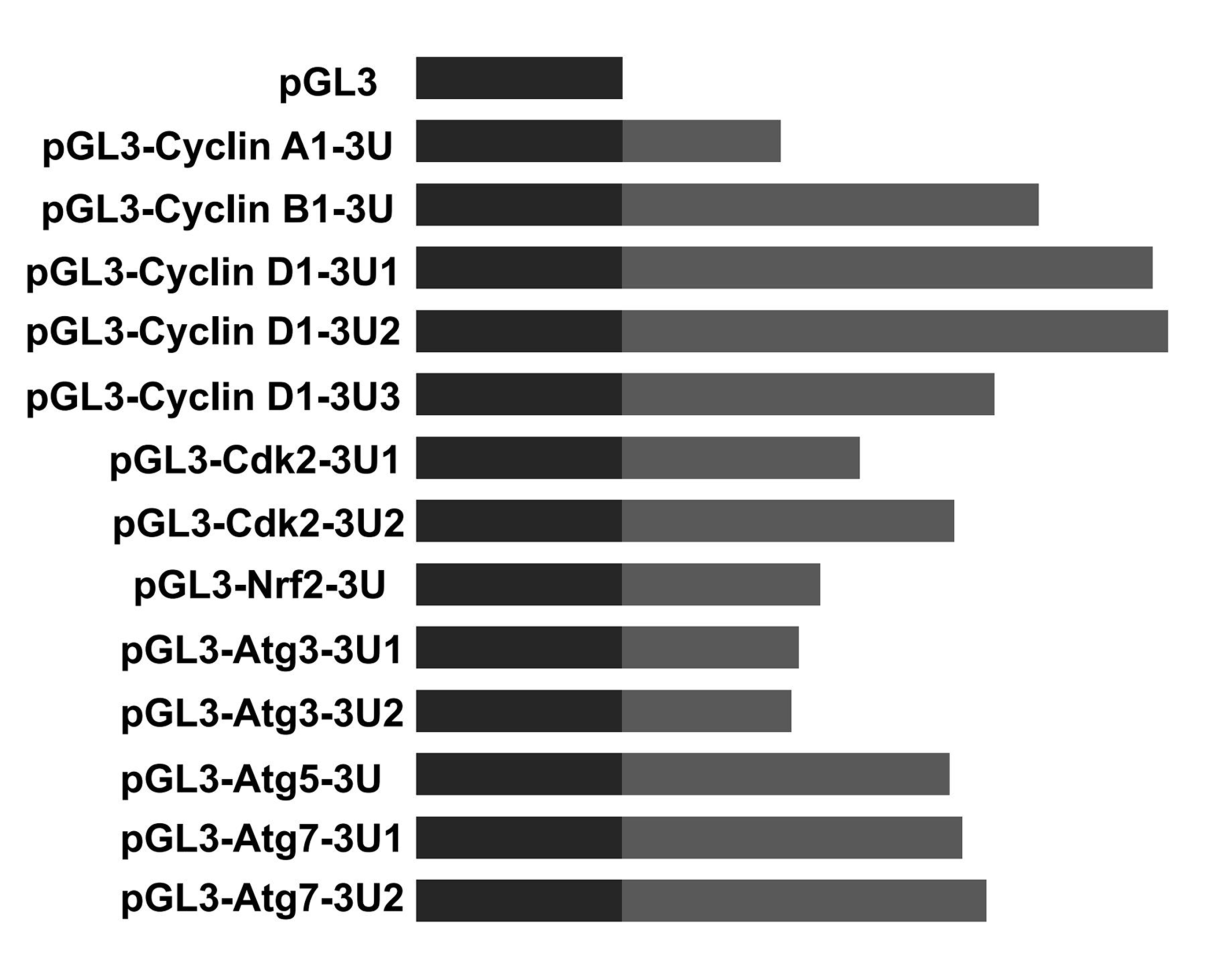


**Supplementary Fig. 10.** Schematics of pGL3-derived reporter plasmids used for double-luciferase reporter assays.

**Supplementary Table 1** Primers used for qPCR.

| Primer | Sequence Size (bp) |
| --- | --- |
| HuR-F:  HuR-R:  Cyp2e1- F:  Cyp2e1- R:  Nrf2- F:  Nrf2- R:  Cyclin A1- F:  Cyclin A1- R:  Cyclin B1- F:  Cyclin B1- R:  Cyclin D1- F:  Cyclin D1- R:  Cdk2- F:  Cdk2- R:  Cdk4- F:  Cdk4- R:  Pcna- F:  Pcna- R:  Atg3- F:  Atg3- R:  Atg5- F:  Atg5- R:  Atg7- F:  Atg7- R:  Atg12- F:  Atg12- R:  P62- F:  P62- R:  Gclc- F:  Gclc- R:  Gclm- F:  Gclm- R:  Nqo1- F:  Nqo1- R:  Ho-1- F:  Ho-1- R:  Sod1- F:  Sod1- R:  Sod2- F:  Sod2- R:  Cat- F:  Cat- R:  18s- F:  18s- R: | TGGGCGAATCATCAACTCC 72  CGGATAAAGGCAACCCCTCT  CGTTGCCTTGCTTGTCTGGA 105  AAGAAAGGAATTGGGAAAGGTCC  CGAGATATACGCAGGAGAGGTAAGA 79  GCTCGACAATGTTCTCCAGCTT  ACCTAAGGCGTCAAGGAGTGT 114  CAGCAACCAAGGAAGGAAGATA  AAGGTGCCTGTGTGTGAACC 228  GTCAGCCCCATCATCTGCG  GCGTACCCTGACACCAATCTC 183  CTCCTCTTCGCACTTCTGCTC  CTCGACACTGAGACTGAAGGT 91  GCAGCTTGACGATATTAGGGTGA  CGTGAGGTGGCCTTGTTAAGGA 74  GCACAGACATCCATCAGCCG  TTTGAGGCACGCCTGATCC 135  GGAGACGTGAGACGAGTCCAT  GTGAAGGCATATCTTCCGACAG 207  CTTGCTTTCCAGTGTAATCTCCT  TGTGCTTCGAGATGTGTGGTT 120  GTCAAATAGCTGACTCTTGGCAA  TGACCTTCGCGGACCTAAAGA 90  CCCGGATTAGAGGGATGCTC  TCGCCCGGAACGGAGGAA 112  TTCGCTCCACAGCCCATTTC  GAGGCACCCCGAAACATGG 79  ACTTATAGCGAGTTCCCACCA  AACACAGACCCACAAACCCAGAG 204  CCGCATCTTCTGGAAATGTT  TGTGTGATGCCACCAGATTT 201  GATGATTCCCCTGCTCTTCA  CAGATCCTGGAAGGATGGAA 202  TCTGGTTGTCAGCTGGAATG  AAGCCGAGAATGCTGAGTTCA 100  GCCGTGTAGATATGGTACAAGGA  AACCAGTTGTGTTGTCAGGAC 140  CCACCATGTTTCTTAGAGTGAGG  TGGACAAACCTGAGCCCTAAG 76  CCCAAAGTCACGCTTGATAGC  TGGCACACTTTGACAGAGAGC 115  CCTTTGCCTTGGAGTATCTGG  CGCCGCTAGAGGTGAAATTCT 101  CGAACCTCCGACTTTCGTTCT |

**Supplementary Table 2** Primers used for PCR.

| Primer | Sequence Size (bp) |
| --- | --- |
| Cyclin A1-3U -F:  Cyclin A1-3U -R:  Cyclin B1-3U-F:  Cyclin B1-3U-R:  Cyclin D1-3U1-F:  Cyclin D1-3U1-R:  Cyclin D1-3U2-F:  Cyclin D1-3U2-R:  Cyclin D1-3U3-F:  Cyclin D1-3U3-R:  Cdk2-3U1-F:  Cdk2-3U1-R:  Cdk2-3U2-F:  Cdk2-3U2-R:  Nrf2-3U-F:  Nrf2-3U-R:  Atg3-3U-F:  Atg3-3U-R:  Atg5-3U1-F:  Atg5-3U1-R:  Atg5-3U2-F:  Atg5-3U2-R:  Atg7-3U1-F:  Atg7-3U1-R:  Atg7-3U2-F:  Atg7-3U2-R:  Ndufb6-5U-F:  Ndufb6-5U-R:  Ndufb6-3U-F:  Ndufb6-3U-R: | (T7)TGGAGCCGCCTGTAGTTC 221  CACAGCCAAACAGCAAGTTG  (T7)CTCCAATAGACTGCTACATCTG 884  CAGCAAATGAGACAGGATTC  (T7)GACATCTGAGGGCCACCG 1013  CATCCCCATCCATTCCATTA  (T7)TAATGGAATGGATGGGGATG 922  GGCATCTGTAAATACACTCT  (T7)TAGAGTGTATTTACAGATGC 712  GAAGCGCCATGGTGTGTCAA  (T7)TGCCCTTCCCAAAGCCC 442  TCTAATTCATTAAAACGGCC  (T7)GGCCGTTTTAATGAATTAGA 692  TTAACAGAACACTGGAGGAC  (T7)GGTTCGGGAGGATGGAGCCT 432  TCTTTTATTAGTGCTAGCTCCT  (T7)TTTGTTCAAGCTGTCATTCC 843  GAGGGATATTCTTAGATGATGT  (T7)GAAAGAGTGTGTCCTCCTCGC 726  CCTGTGACAGCTCCTGAGAC  (T7)GTCTCAGGAGCTGTCACAGG 478  TTAGCATACTCAGATGGGTTG  (T7)GAAGCAAGCAACCACAGC 753  TCTGTGTTAGGCCAAGGTC  (T7)GACCTTGGCCTAACACAGA 829  AAGACCATCATTACGCTGTGC  (T7)GCGCCGCTTCCAGGCGCC 96  TACCCTGACATGTTGCCG  (T7)GATCAACATCATTGAAGAG 171  CAAATCATAGAACCTTTGGACAG |

**Supplementary Table 3** Primers used for PCR.

| Primer | Sequence Size (bp) |
| --- | --- |
| Cyclin A1-3U -F:  Cyclin A1-3U -R:  Cyclin B1-3U-F:  Cyclin B1-3U-R:  Cyclin D1-3U1-F:  Cyclin D1-3U1-R:  Cyclin D1-3U2-F:  Cyclin D1-3U2-R:  Cyclin D1-3U3-F:  Cyclin D1-3U3-R:  Cdk2-3U1-F:  Cdk2-3U1-R:  Cdk2-3U2-F:  Cdk2-3U2-R:  Nrf2-3U-F:  Nrf2-3U-R:  Atg3-3U1-F:  Atg3-3U1-R:  Atg3-3U2-F:  Atg3-3U2-R:  Atg5-3U-F:  Atg5-3U-R:  Atg7-3U1-F:  Atg7-3U1-R:  Atg7-3U2-F:  Atg7-3U2-R: | CCCTCTAGATGGAGCCGCCTGTAGTTC 221  CCCTCTAGACACAGCCAAACAGCAAGTTG CCCTCTAGACTCCAATAGACTGCTACATCTG 884  CCCTCTAGACAGCAAATGAGACAGGATTC CCCTCTAGAGACATCTGAGGGCCACCG 1013  CCCTCTAGACATCCCCATCCATTCCATTA CCCTCTAGATAATGGAATGGATGGGGATG 922  CCCTCTAGAGGCATCTGTAAATACACTCT CCCTCTAGATAGAGTGTATTTACAGATGC 712  CCCTCTAGAGAAGCGCCATGGTGTGTCAA  CCCTCTAGATGCCCTTCCCAAAGCCC 442  CCCTCTAGATCTAATTCATTAAAACGGCC  CCCTCTAGAGGCCGTTTTAATGAATTAGA 692  CCCTCTAGATTAACAGAACACTGGAGGAC  CCCTCTAGAGGTTCGGGAGGATGGAGCCT 432  CCCTCTAGATCTTTTATTAGTGCTAGCTCCT  CCCTCTAGATTTGTTCAAGCTGTCATTCC 360  CCCTCTAGAGCTAGTTCTGACACTGGTAG  CCCTCTAGAAAGTGTTCCATGCCAGCCT 477  CCCTCTAGAGAGGGATATTCTTAGATGATGT  CCCTCTAGAGAAAGAGTGTGTCCTCCTCGC 1006  CCCTCTAGAGACAGGTCACCTGCAGGCT  CCCTCTAGAGAAGCAAGCAACCACAGC 753  CCCTCTAGATCTGTGTTAGGCCAAGGTC  CCCTCTAGAGACCTTGGCCTAACACAGA 829  CCCTCTAGAAAGACCATCATTACGCTGTGC |
